# Beyond Oxidation: Engineering Functional Anodised Metal Matrices Through Molecular and Surface Modifications

**DOI:** 10.3390/ijms26167809

**Published:** 2025-08-13

**Authors:** Mateusz Schabikowski, Agnieszka Stróż, Andrzej Kruk

**Affiliations:** 1 Institute of Nuclear Physics Polish Academy of Sciences, Radzikowskiego 152, 31-342 Kraków, Poland; 2 Institute of Materials Engineering, University of Silesia, 75 Pułku Piechoty 1A, 41-500 Chorzów, Poland; agnieszka.stroz@us.edu.pl; 3 Faculty of Space Technologies, AGH University of Kraków, al. A. Mickiewicza 30, 30-059 Kraków, Poland

**Keywords:** anodisation, chemical functionalisation, functional materials

## Abstract

Anodised metal matrices represent a versatile and multifunctional platform for the development of advanced materials with tunable physicochemical properties. Through electrochemical oxidation processes—commonly referred to as anodisation—metals such as aluminium, titanium, niobium, zinc and tantalum can be transformed into structured oxide layers with defined porosity, thickness and surface morphology. These methods enable the fabrication of ordered nanoporous arrays, nanotubes and nanowires, depending on the process parameters and the type of metal. The review introduces and outlines the various anodisation techniques and parameters. This is crucial, since each individual metal requires specified optimal conditions to obtain a stable anodised oxide layer. This review provides an overview of recent advances in the design and application of anodised metal substrates, with the focus on their role as functional platforms in catalysis, sensing, energy storage and biomedical engineering. Special attention is given to post-anodisation surface modification strategies, such as chemical functionalisation, thin-film deposition and molecular-level integration, which significantly expand the utility of these materials. The review also highlights the challenges, limitations and future perspectives of anodising technologies, aiming to guide the rational design of next-generation devices based on engineered oxide architectures.

## 1. Introduction

The anodising process, originally developed as a method of increasing the surface corrosion resistance of metals, especially aluminium, has undergone significant evolution since its introduction in the first half of the 20th century. This research was initiated by Gunterschulze and Betz [1], Cabrera and Mott [2], Young [3], Vetter and Wagner [4] and subsequently continued by many other authors. Research on the electrochemical oxidation of metals has provided a consistent picture of the kinetics and mechanism of formation and structure of oxide layers on metals. Initially, this technique involved the electrochemical production of thin oxide layers in simple electrolytes based on sulphuric, phosphoric or chromic acid. The anodisation of metals and alloys is currently the subject of intensive research due to the diverse and unique applications of oxide layers in modern technology [3,5].

Currently anodic oxidation is a popular method for obtaining oxide nanostructures on metals and their alloys. It is a process in which a homogeneous layer is formed on the metal surface, which is characterised by very good properties. anodising usually increases the thickness and density of the oxide layer on the metal surface. To produce it, the conductive element is connected to the positive terminal of a direct current source. It acts as an anode in an electrolytic bath. In this case, the cathode can be a platinum rod or plate. When voltage is applied, electrons are drawn toward the positively charged anode. As a result, the exposed metal atoms react with the oxygen ions contained in the electrolyte. The main advantages of metal anodising include its versatility. The process allows for the shaping of the structure’s morphology and chemical composition. The reaction results in the formation of an integral oxide layer. Another important aspect is the fact that anodising allows for precise control of the properties of oxide layers. This is achieved by selecting process conditions such as time, voltage and electrolyte composition [6].

There are three different methods of anodic oxidation of metals:

- galvanostatic method (at a constant current density),
- potentiostatic method (at a constant potential),
- combined method (initial oxidation at a constant current.

When the electrode potential reaches the desired value, the process switches to potentiostatic conditions) [5,6].

The morphology and properties of oxide layers depend on the process parameters. These include voltage, the electrolyte, temperature and oxidation time [3,7].

The technique of anodic oxidation is based on placing the workpiece in the electrolyte and passing through the system material (electrode)-electrolyte current with the set parameters.

Anodisation has emerged as a robust technique for the fabrication of highly organised oxide matrices that exhibit unique structural and functional properties. The process is a result of extending the natural tendency of oxidation of metals when exposed to air. Such idle passivation progresses until the formed layer becomes too difficult for ions to traverse through, thereby preventing the oxidation reaction with the metal substrate from continuing. Thus, naturally formed layers typically have a thickness of 5–10 nm. The driving force for the ions to penetrate larger layers can be increased by changing the medium to a liquid (and thus increasing the concentration of ions), applying an electrical potential to the metal substrate and, finally, utilising a medium that dissolves the forming oxide. This leads to the formation of channels in the oxide layer which allows ions to have continuous access to the metal reagent. Concurrently, the oxide is formed on the sides of the channel, building a tower-like structure with an empty cylindrical passage in the middle. Such structures are formed periodically in radial arrangement from the first channel. In ideal conditions, the pores (channels) are arranged in hexagonal order, utilising the densest packing.

Over the decades, anodisation—particularly of aluminium—has been the subject of extensive research and numerous review articles. Wood reviewed the sealing process of anodised aluminium oxide (AAO) as early as in 1959 [8]. Lee and colleagues described the influence of anodisation parameters on pore morphology [9], while Sarkar and co-workers and co-workers examined AAO as a template for nanowire synthesis [10]. Minagar et al. reviewed applications in biocompatible implants [11] and Poinern et al. addressed AAO in tissue engineering [12]. Other reviews have explored AAO as a cell interface [13], as a platform for sensing [14,15] or in general discussions of fabrication and characterisation techniques [16,17,18,19,20,21,22,23]. Fernández-González et al. discussed patterning of ultra-thin aluminium oxide films and emphasised their relevance in scalable top-down manufacturing [24]. The historical review by Tajima [25] remains, in our opinion, a cornerstone reference in the field.

Del Olmo and colleagues reviewed anodisation in steels and iron-based alloys [26]. In a different direction, anodisation has also been employed in the synthesis of intermetallic compounds such as Ti–Cu [27]. Moreover, anodic oxidation processes have proven effective in environmental applications, such as the degradation of pharmaceutical contaminants in water using specialised electrodes [28].

While these reviews offer comprehensive insights into various aspects of anodisation—from morphology control to biomedical and environmental applications—relatively few have focused on the chemical functionalisation of anodised materials. Yet, surface modification is a crucial factor that defines how these materials interact with their environment and determines their suitability for advanced applications, such as catalysis, biosensing or selective adsorption.

Importantly, anodisation is not limited to aluminium. Its inherent versatility allows it to be applied to a wide range of metals, including aluminium, titanium, niobium, zinc, copper, magnesium, tantalum and iron, each yielding oxide layers with distinct morphologies, chemistries and functionalisation opportunities. This breadth of applicability significantly expands the technological potential of anodised materials across fields as diverse as biomedicine, energy storage and environmental remediation.

In this context, the present review aims to fill a critical gap by focusing on the chemical functionalisation of anodised surfaces across different metals. We highlight recent advances in surface modification strategies, functionalisation chemistries and resulting material properties—demonstrating how tailored anodic layers can be leveraged far beyond their traditional structural or protective roles. By offering a cross-material perspective on functionalisation, this article provides an up-to-date and application-oriented outlook that complements the existing literature and underscores the full potential of anodisation as a platform for advanced material design.

## 2. Fundamentals of Anodisation

To illustrate the principles of anodisation, we focus on the example of titanium—a metal whose anodised oxide layers often adopt the form of vertically aligned nanotube arrays. These one-dimensional nanostructures display unique properties compared to conventional, continuous ultrathin oxide films [29,30]. Studies have shown that the presence of such nanotubular anodic layers on titanium surfaces enhances osteoblast adhesion and proliferation, while also improving corrosion resistance and bioactivity.

One of the key advantages of anodisation over other methods for producing oxide nanotube layers lies in its high degree of tunability. The morphology, structure and geometry of the resulting nanotubes can be precisely tailored by adjusting the parameters of the electrochemical oxidation process. For instance, the applied anodisation voltage directly influences the inner and outer diameters of the nanotubes, while the duration of the process determines their average length. Additionally, the composition of the electrolyte—particularly its viscosity, pH and fluoride ion concentration—plays a crucial role in modulating oxide dissolution rates and overall reaction kinetics [3,7,29,31,32,33].

Nanotubular oxide structures can be produced by anodisation on the surface of metals that exhibit self-passivation. These include titanium, niobium and zirconium and other transition metals [32,34].

The electrolyte solution for anodising usually contains fluoride ions from HF or NH_4_F. Crawford et al. [35] proposed a mechanism for anodising titanium in electrolytes containing F^−^ ions, in which three successive stages occur. In the first step, when an appropriate voltage or current density is applied to the anode, oxidation of the titanium (reaction (1)). As a result of the reaction of Ti^4+^ ions with OH^−^ and O^2−^ ions, a thin and continuous layer of TiO_2_ is formed on the surface of the anode (reaction (2)):

(1) $$\mathrm{Ti}\to {\mathrm{Ti}}^{4+}+4e$$

(2) $${\mathrm{Ti}}^{4+}+2H_{2}O\to {\mathrm{TiO}}_{2}+4H^{+}$$

The growth of the TiO_2_ layer is supported by an electric field and occurs as a result of the migration of Ti^4+^ ions through the resulting oxide layer toward the electrolyte and the transport of O^2−^ ions toward the anode surface. The first stage lasts only a few to a few tens of seconds and is characterised by an exponential decrease in anodisation current density, which is caused by an increase in the thickness of the barrier TiO_2_ layer [7]. In the second stage, F^−^ ions adsorbed on the surface of the oxide layer migrate deep into it, which causes local dissolution of the TiO_2_ layer and consequent formation of irregular pores. The result of the second stage is the formation of insoluble complexes of [TiF_6_]^2−^ (reaction (3)).

(3) $${\mathrm{TiO}}_{2}+6\mathrm{HF}\to {\left[{\mathrm{TiF}}_{6}\right]}^{2-}+2H_{2}O+2H^{+}$$

**Figure 1:**
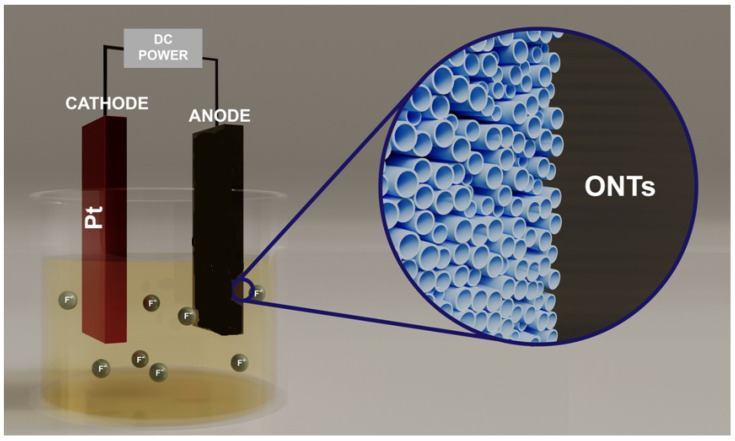
The anodisation scheme of titanium.

In fluoride-free solutions, both the thickness of the barrier layer and the measured current density reach a steady state. In electrolytes containing fluoride ions, a nanoporous structure begins to form as a result of the chemical dissolution of the barrier oxide layer (reaction (3)). In the second stage, there is a slight increase in the value of the anodisation current density in the presence of F^−^ ions, which is due to a decrease in the thickness of the TiO_2_ layer. The pores formed begin to branch, overlap and compete for the available current. In the third stage, after stabilizing the value of the anodisation current density, the nanotube matrix is formed under optimal current-voltage conditions, in which the current is distributed evenly among the pores leading to self-organization of the porous layer. Further anodisation increases the length of the nanotubes and the anodic current density does not significantly affect the structure of the resulting TiO_2_ nanotube layers [3,35,36,37]. A scheme of the anodising process is shown in Figure 1. The formation of TiO_2_ nanotubes in electrolytes containing F^−^ ions is thus the result of two competing electric field-assisted processes, namely the hydrolysis of Ti with the formation of TiO_2_ (reaction (2)) and the chemical dissolution of TiO_2_ at the oxide/electrolyte interfacial interface (reaction (3)), resulting in the formation of [TiF_6_]^2−^ (reaction (3)). The process of TiO_2_ nanotube growth proceeds through the formation of an initial barrier layer, the formation of evenly spaced pores and the separation of the interconnected pores into nanotubes. A detailed understanding of the exact mechanisms of formation of ordered oxide nanotubes is still not clear [35]. The choice of the electrolyte in which the anodisation process is carried out has the greatest impact on the microstructure and properties of the oxide nanotube layers obtained. Over the past decade, four generations of oxide nanotubes obtained on the surface of titanium and its alloys have been distinguished [38]. For the first time, a layer of oxide nanotubes on the surface of titanium was obtained by anodisation in 1999 by Zwilling et al. [39], who produced nanotubular structures using an aqueous electrolyte containing hydrofluoric acid and chromic acid. The TiO_2_ layers obtained under these conditions were inhomogeneous and had a small length of about 500–600 nm due to the high rate of chemical dissolution of titanium dioxide in HF solution. Gong et al. [40] showed that a titanium electrode anodised in a 0.5% HF solution at 20 V for 6 h has the same thickness as an electrode anodised for only 20 min under identical conditions.

**Figure 2:**
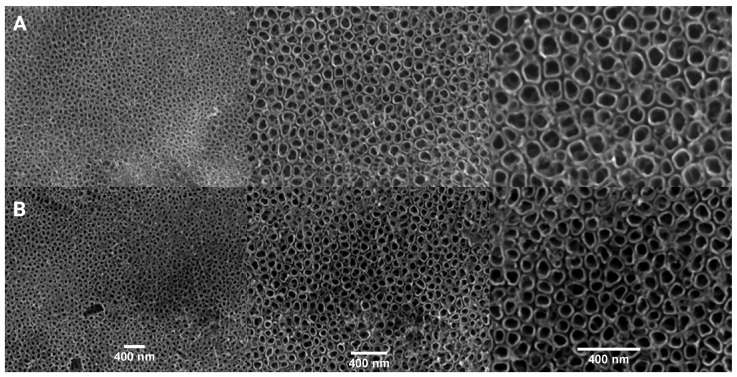
Anodised titanium oxide fabricated in 0.8% HF solution for 30 min; row (**A**): 15 V, row (**B**): 20 V. Scale bars shown at the bottom of each column apply to all images in that column.

Currently, the first-generation layers are obtained from aqueous electrolytes that contain hydrofluoric acid or its salt in the amount of 0.1 to 1 wt% or using mixtures of HF with other acids, such as HNO_2_ + HF, HNO_3_ + HF, H_2_SO_4_ + HF, H_2_Cr_2_O_7_ + HF or H_3_PO_4_ + HF with a concentration of no more than 1 M [41]. The first generation nanotubes formed on titanium are shown in Figure 2.

Highly ordered layers of second–generation oxide nanotubes with nanotube lengths of up to 2–3 μm can be produced from aqueous buffer solutions with varying pH values, which contain the addition of fluorine salts in the form of NaF, KF or NH_4_F instead of HF in an amount of about 0.5 wt% [42]. The use of such selected electrolytes is intended to slow down the rate of chemical dissolution of the oxide layers, which occurs rapidly in acid solutions so that nanotube growth is limited. The concentration of fluoride ions determines the dissolution rate and must be kept as low as possible, but high enough to ensure the growth of nanotubes, since the concentration of F^−^ ions also affects the pH of the solution. The most commonly used electrolytes for obtaining second-generation oxide nanotubes are mixtures of 1 M Na_2_SO_4_ with 0.5 wt% NaF and 1 M (NH_4_)H_2_PO_4_ with 0.5 wt% NH_4_F [43].

The optimal pH value of such solutions is 3–5. At pH values above 5, increased hydrolysis of titanium ions occurs during anodisation of titanium and its alloys and deposition of a mixture of titanium hydroxides on the surface of oxide nanotube layers is observed, which is difficult to remove even with an ultrasonic cleaner.

Layers of third-generation oxide nanotubes are obtained from electrolytes containing polar organic solvents such as formamide, N-methylformamide, ethylene glycol (EG), diethylene glycol, dimethylsulphoxide, methanol and glycerol, along with the addition of a fluorine ion source in the form of 1–6 wt% HF, 0.6 wt% NH_4_F or quaternary ammonium salts of fluorine and a small amount of H_2_O (1–5%) [44]. The water content of aqueous electrolytes is responsible for the dissolution rate of the top surface of oxide nanotubes, so reducing the amount of water in these electrolytes increases the length of the resulting nanotubes up to 1000 μm. For some electrolytes containing glycerol or methanol, higher amounts of H_2_O (25–50%) are used. Glycerol-based electrolytes containing different amounts of H_2_O are characterised by varying viscosity, which allows shaping the morphology and properties of the obtained nanotubular oxide films. It has been shown that third-generation oxide nanotubes with very smooth walls can be obtained from electrolytes containing ethylene glycol [44].

Fourth-generation oxide nanotubes are obtained using the recently developed rapid breakdown anodisation technique from electrolytes that do not contain fluoride ions, mainly from an aqueous solution of 0.15 M HCl or a mixture of 0.5 M HCl and 0.1–0.4 M H_2_O_2_ [45,46]. Fourth-generation oxide nanotubes are typically up to several hundred nanometers in length and are produced in a very short time of a few minutes [47]. Anodisation of titanium in electrolytes that were a mixture of 0.4 M NH_4_Cl and 0.5 M HCl, H_2_SO_4_, C_2_H_2_O_4_, CH_2_O_2_, CCl_3_COOH or C_6_H_12_O_7_ was also carried out [48]. The length of the produced thin oxide nanotubes with insufficient adhesion to the substrate reached up to 60 μm, as in the case of using an electrolyte containing 0.1 M HClO_4_ [46,49].

It is worth noting that the geometry of nanotubular oxide films during the anodisation process can be modified differently by changing the voltage. It is possible to obtain geometries such as stacks of nanotubes, bamboo nanotubes, nanorods, nanotubes with branching or double-walled nanotubes [3].

## 3. Anodisation of Different Metals: Chemistry and Process Optimisation

### 3.1. Aluminium

Aluminium has become more and more popular in the 20th century as a competing material for iron and copper. It is approximately three times lighter than those but it lacks in mechanical strength and corrosion resistance. The smaller weight of components would greatly improve the fuel efficiency of vehicles made of them. This was the main driving force to improve upon the lacking parameters of such a promising metal. The first approach to solve that issue was to combine them with other elements forming alloys that improved weldability, strength and corrosion resistance. A different attempt was to form an oxide layer which greatly improved those parameters. Moreover, the outstanding structure of the oxide layer was exploited even more with the industrial process of colouring aluminium parts—dyes would be inserted into the pores of anodised aluminium oxide and covered with a transparent layer. Such an arrangement provides a remarkable improvement in colour retention because the paint is not in direct contact with the environment and users. The outstanding microstructure of anodised aluminium has also been noticed for its great potential in surface engineering.

The first observation of the electrochemical oxidation of aluminium was made by Sir Charles Wheatstone in 1855 [50]. During the process of classification of aluminium in the voltaic series, he observed a minor “action” of the metal in nitric acid and a small electrical current in sulphuric acid. He concluded his observation with:

“It is rather remarkable, that a metal, the atomic number of which is so small and the specific gravity of which is so low, should occupy a position in the electromotive scale as to be more negative than zinc in the series.”

Two years later, Buff et al. reported aluminium to become anodic when coupled with platinum in sulphuric acid [51]. Several decades later, Benegough and Stuart secured a patent pertaining to the industrial-scale production of anodised aluminium and its alloys [52] opening the way for a more common application of anodisation of aluminium for protective purposes.

Keller and co-workers described anodised aluminium oxide as a layer made of hexagonally arranged pores and separated the porous and barrier layers [53]. This theory is commonly accepted in a more or less unchanged form to this day.

**Figure 3:**
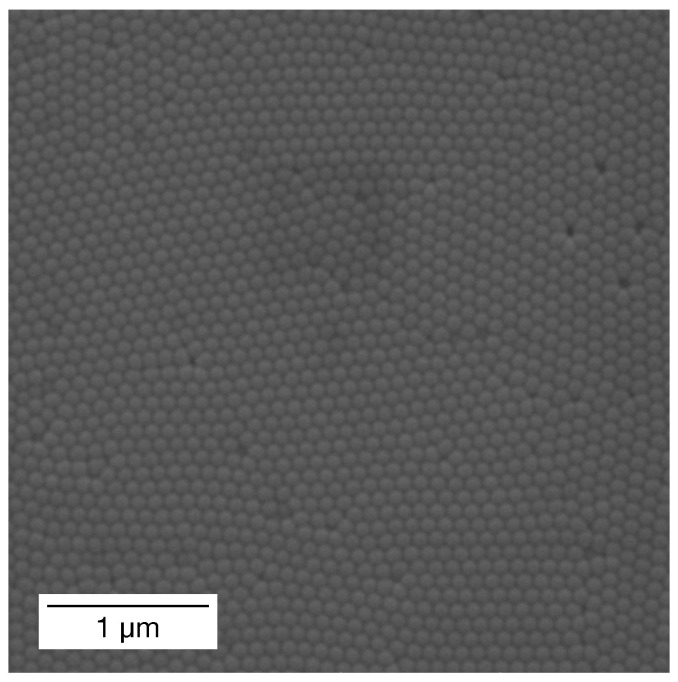
A bottom layer of an anodised aluminium oxide synthesised from a single-crystal aluminium at 40 V in 0.3 M C_2_H_2_O_4_·2H_2_O at 12 °C.

For many applications, the hexagonal arrangement of the pores close as close to ideal as possible is required. Because anodisation is sensitive to all kinds of fluctuations, such as impurities in the substrate, changes in temperature and concentration of the electrolyte or uneven surface of an anode, obtaining such an arrangement is not a trivial matter. While using a high-purity substrate, ensuring a proper cooling and mixing systems or polishing substrates before the process may significantly improve the final ordering, the array may still suffer disturbances like in this case of anodisation of single-crystal aluminium plate with active cooling and mixing (Figure 3).

One of the commonly used methods to improve the arrangement is a so-called two-step anodisation process developed in 1995 by Masuda and Fukuda [54]. Masuda and colleagues started working on refining the arrangement of alumina pores already in 1990 [55]. However, as the authors say in [54]:

“In our early work (16), the regularity of the nanohole array in the replicated structure was unsatisfactory; this resulted from imperfections in the cell arrangement in the mother anodic porous alumina used as a starting material.”

Their method consists of two anodisation processes of the same substrate with the removal of the anodised oxide in between them. Typically the first anodisation is shorter since its desired leftover products are the indentations on the metallic substrate. The pores forming during anodisation are open on the electrolyte side and closed on the substrate side with a hemispherical shape at their bottom–the barrier layer. Because the metal tightly adheres to the oxide layer, the convex hemispherical shape of the barrier layer is imprinted in the substrate producing a mesh of U-shaped indentations. After chemically removing the anodised layer formed during the first anodisation, the substrate is preprinted with a non-ideal array of indentations. Those indentations are starting points for more ordered pores formed during the second (and proper) anodisation because of their less chaotic locations.

Masuda and Satoh [56] used such prepared highly ordered AAO matrix as a mask to deposit 50-nm thick gold nanodots of approximately 40 nm in diameter over a 2 mm × 2 mm area on a single-crystal silicon. They wanted to use it as a mask for reactive ion etching to fabricate semiconductor devices on a nanometer scale. That was one of the reasons for producing highly ordered pores in the AAO matrix.

**Figure 4:**
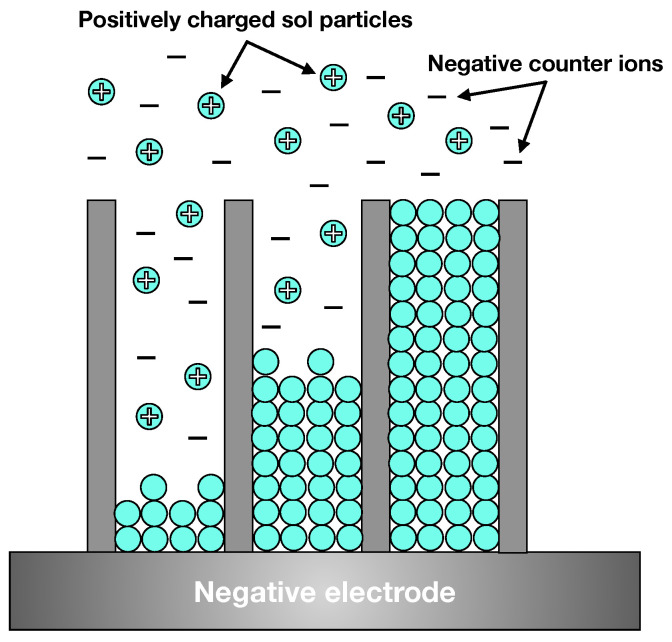
The timeline of pore filling in the forced electrophoresis deposition. Based on the work of Limmer et al. [57].

One of the outstanding features of anodised materials is their porous structure. In order to give matrices new functionality, a different material can be inserted into the pores. However, even with pores in the range of tenths of nanometers, it is not a trivial task to achieve complete filling. One of the ways to improve it is to build a system with an electric field between a matrix and another electrode in an electrolyte and force electrophoresis of the desired material into the pores as shown in Figure 4.

A typical limitation of this method is the electric potential threshold at which the electrolyte decomposes. Despite the obstacles, Caubert and others [58] successfully used pulsed electrophoretic deposition of boehmite (AlOOH) nanoparticles inside pores of anodised aluminium alloys. The pulsed character of the process is one of the key factors in limiting the unwanted electrolysis of an electrolyte and making it possible to use water as the medium. Gonçalves et al. [59,60] extended this work and used a similar process to deposit silanes from organic and inorganic precursors. The group explored this functionalisation mainly in the field of hydrophobicity and sealing performance of the membranes.

Miller and Majda functionalised anodic aluminium oxide with ferrocene ions for electrical conductivity [61]. This was realised by depositing poly(4-vinyl-pyridine), PVP, on the walls of AAO pores leaving their centres open. PVP functions as a molecular anchor for ferrocene ions and its deposited amount determines the concentration of iron ions.

This success encouraged the scientists to further enhance molecular immobilisation on the outstanding surface of anodic aluminium oxide. The same authors, relying on molecular self-assembly, deposited a bi-layer consisting of a silane, which is often used as a bridge between ceramic and organic components and a surfactant [62]. A molecular monolayer of *n*-octadecyltrichlorosilane was first deposited by simple immersion of the membrane in the silane solution for 15 min. This made the AAO surface hydrophobic. In the next step, the deposition of *N*-methyl-*N′*-octadecylviologen was realised by exposing the treated membrane to its aqueous solution. The anodic membranes used in the experiments had an average pore size of 100 nm. The calculated porosity at each step of the process was 51 ± 3%, 44 ± 3% and 41 ± 3% for pure, silanised and final membranes respectively. Thus, the pores retained their permeability with completely changed surface characteristics.

Brumlik and co-workers used molecular functionalisation of anodic aluminium membranes to form hollow gold tubes inside pores of the template [63,64]. The scientists used AAO pores as a template for the tubes to grow. However, a simple electrodeposition of a metal on an anodised aluminium oxide matrix would likely result in filling the pores forming wires. Having the idea of creating hollow metallic tubes inside the pores, the authors functionalised the surface of the pores with molecular anchors in the form of a silane. To attach gold ions to the walls, 0.1 vol% 2-cyanoethyltriethoxysilane in anhydrous hexadecane was used because of its strong affinity to the metal. Remarkably, the process was realised by sonication in a mere 30 s. After an overnight treatment at 100 °C, a 20-nm layer was sputtered on the membrane to act as a cathode to the gold electroplating that followed it. As a result, an AAO membrane with partly filled hollow gold tubes was formed. The tubes are connected on one side of the membrane via a gold layer. The authors reported that tubes of up to 2 μm can be formed with the method. Dissolution of the AAO membrane in hydrofluoric acid exposes the free-standing parallel tubes. Individual tubes can also be collected via filtration by first dissolving the bottom gold layer in aqua regia and subsequent dissolution of the membrane.

In order to extend the length of gold tubes, the authors utilised flow-assisted electrolysis. The process is analogous to the previous one with the exception of using a peristaltic pump to force the solution through the pore via pressure. This modification extended the length of the tubes to 3 μm.

Zhang and co-workers chemically deposited NiCoAl layered double hydroxides on anodised aluminium oxide to enhance its corrosion resistance [65]. Moreover, the layer provides superhydrophobic and self-cleaning properties to the material further improving its functionality.

Another approach to the chemical modification of anodised aluminium oxide matrices is doping. Poznyak and colleagues realised doping by using aluminium alloys as the substrate for anodisation [66]. This way the doped elements were building in the AAO structure already during the formation.

### 3.2. Titanium

TiO_2_ nanotubes can be synthesised using a hard matrix, hydro/solvothermal, electrospinning and anodic oxidation methods to obtain the material in powder or thin film form [29]. Particularly interesting from the application point of view are TiO_2_ nanotube layers obtained by anodic oxidation. This method shows a number of advantages over other methods, namely the following: low process temperature (usually ambient) is required, along with low cost of reagents and apparatus, mild reaction environment, ease of process control, short synthesis time and no deposition step—nanotube layers grow on a titanium substrate. The resulting materials are in the form of a thin film [30]. The first layer of TiO_2_ nanotubes with a thickness of about 500 nm was obtained in an electrolyte composed of chromic acid and hydrofluoric acid [29,38]. Subsequently, the use of an aqueous acidic electrolyte led to the preparation of TiO_2_ nanotubes with rough walls and lengths reaching 1 μm. Highly ordered layers of TiO_2_ nanotubes were obtained by further improving electrolyte compositions (using glycerol, ethylene glycol, DMSO) and controlling the anodic oxidation process (voltage, time) [3,38]. Research on titanium(IV) oxide nanotubes obtained by anodic oxidation led to the development of the fourth generation of TiO_2_ nanotube films:

- Generation I—obtained in inorganic aqueous electrolytes (mainly based on HF or HF + H_3_PO_4_), 200–500 nm,
- Generation II—obtained in buffered electrolytes (Na_2_SO_4_ + NaF or (NH_4_)_2_SO_4_ + NH_4_F),
- Generation III—obtained in the presence of organic electrolytes containing fluoride ions (NH_4_F + H_2_O + glycerol or NH_4_F + H_2_O + ethylene glycol),
- Generation IV—obtained in electrolytes that do not contain fluoride ions (mainly HClO_4_, NaCl and H_2_O_2_).

The use of TiO_2_ nanostructures is usually due to the exploitation of some unique feature of TiO_2_ (e.g., ionic, electrical or biocompatibility) and significant enhancement of certain reaction or transport rates, which are achieved by using small size (large surface area, short diffusion path) or size-limiting effects [38].

Due to their one-dimensional morphology and thus unique properties, TiO_2_ nanotubes have found applications in many areas: heterogeneous photocatalysis, dye-sensitised photovoltaic cells, electrochromic devices, lithium-ion batteries, sensors, supercapacitors and biomedicine. Among various semiconductor materials, TiO_2_ has found the widest application in photocatalytic reactions [34,44]. It is used for the degradation of pollutants in the gas and aqueous phases, generation of hydrogen, photoconversion of CO_2_ to useful hydrocarbons or transformation of organic compounds [3]. Macak et al. [36] showed that TiO_2_ nanotubes have higher photocatalytic activity compared to TiO_2_ nanoparticle films. Since then, there has been a surge of interest in the application of TiO_2_ nanotubes in photocatalytic reactions. Among other things, studies have been conducted to determine morphological (including length, diameter, wall thickness, single wall, double wall) and structural (including crystallite size, anatase to rutile ratio) parameters on photocatalytic activity [30]. TiO_2_ nanotubes have also undergone various modifications to improve the UV properties of this material, but also to extend its activity into the visible range. Among these, modifications can be distinguished as (a) doping with non-metals and metals, (b) deposition of noble metal nanoparticles and quantum dots, (c) formation of semiconductor heterojunctions and (d) formation of organic–inorganic hybrid systems [3].

Surface modification resulting in a change in physical properties also entails a change in the chemical properties of the surface. The implant interacts with the surrounding tissues through its surface. This interaction is related to the interaction of biological fluids with the implant surface. This interaction is often mediated by proteins absorbed from the biological fluids. The surface features regarding its roughness, topography and surface chemistry are then “translated” by the protein layer into information that cells can understand. The presence of oxygen-containing functional groups such as carbonyl, carboxyl and ester groups increase the wettability and polarity of the surface which facilitates the adsorption of adhesion-mediating proteins including fibronectin, as well as extracellular matrix proteins, resulting in increased cell adhesion. In addition, hydrophilic surfaces, i.e., rich in oxygen groups, provide such a spatial distribution of adsorbed proteins that further promotes easier cell adhesion. On the other hand, a lower amount of oxygen groups changes the surface properties towards more hydrophobic which in turn stimulates adsorption of non-adhesive protein molecules such as albumin. Hydrophobic properties also increase with increasing bicarbonate content on the material’s surface [44,67].

Cell adhesion occurs more easily on surfaces with a positive charge. This is related to the fact that molecules that promote cell adhesion have a positive charge. Positively charged NH^4+^ amino groups therefore promote cell adhesion. The negative surface charge associated with the presence of functional groups such as carboxyl groups -COOH and sulphur groups impairs both cell-material adhesion and the adhesion of cells to each other. Anselme, on the other hand, reports that protein adsorption is improved by negative charge [67].

### 3.3. Other Metals

Over the past two decades, anodising techniques have been employed to fabricate nanostructured surfaces and bulk samples. Anodisation is an electrochemical technique commonly used to modify the surface of niobium through the formation of a metal oxide layer. This method is considered simple and cost-effective, as it does not require technologically advanced or expensive equipment. Anodisation is performed most commonly on high-purity aluminium foil (>99.99%), which serves as a standard substrate [68]. However, anodising lower-purity aluminium alloys, such as AA1050 (approximately 99.5%), has been reported in a number of studies [69,70,71,72,73,74,75,76,77,78,79,80]. From an economic standpoint, cheap Al steel presents a highly attractive alternative due to its significantly lower cost—up to a thousand times cheaper than high-purity aluminium. Nevertheless, the fabrication of anodic aluminium oxide membranes from low-purity Al is nontrivial and typically necessitates carefully optimised conditions that differ from those used in the anodisation of pure aluminium. Scientists are looking for cost-saving solutions through deliberate doping or using cheaper matrices containing certain amounts of other elements. Alternative AAO materials include nanosize porous and tubular matrices modified with doped elements such as niobium [81], zinc [82], iron [83,84], cobalt [9], tantalum [85] iron [83]. However, limited research has been conducted on the development of porous oxide nanostructures in AAO other than Ti. Intentional or accidental doping (or by contamination) materials find diverse applications in fields such as catalysis [86,87,88,89,90], photocatalysis [91,92,93], thermal protection [94], supercapacitors [95], photoelectrodes [96], etc. Impurities, including defects, arise from substrate irregularities, contamination within the deposition chamber and inconsistencies during the film growth process [97]. Various types of defects have been identified in physically deposited thin films, with nodular and trough defects being the most frequently reported [98,99]. These imperfections compromise the film’s ability to effectively shield the underlying substrate from corrosion.

Although this review focuses in detail on selected valve and base metals (Al, Ti, Nb, Ta, Fe, Zn, Cu, Mg), other metals such as lead and tin can also undergo anodic oxidation. The anodisation of these metals has been reported in the literature, with studies exploring the formation of PbO_2_ layers and structures under specific electrochemical conditions. However, because this review focuses on metals with broader applications—and given that lead is increasingly regulated due to its toxicity and environmental impact, while tin oxide (SnO_x_), compared with TiO_2_, exhibits higher electron recombination rates and lower conversion efficiency—these materials are not examined in detail here. For completeness, they are briefly discussed below.

Research on the electrochemical oxidation of lead began as early as 1955, when Fleischmann and Thirsk observed the formation of lead sulphate and its subsequent conversion to lead(IV) oxide in sulphuric acid under constant voltage conditions [100]. This work was later extended by the original authors [101] and independently by Burbank [102,103], as well as by Rüetschi and Angstadt [104,105]. Despite lead being generally discouraged for use, researchers continue to examine its properties in various applications, including a 2025 study on wastewater depollution in the textile industry [106]. Alternative, the authors employ surface treatment techniques on the positive plate grid of lead-acid batteries to develop a stable, capacitive gradient oxide film, aiming to enhance battery capacity and prolong operational lifespan. The manuscript investigates the deposition mechanism of *α*-PbO_2_ on Pb-Ca-Sn alloy using various electrochemical techniques, including anode polarization, galvanostatic polarization and steady-state polarization analyses [107].

Tin oxide, an n-type semiconductor, remains a promising candidate for gas sensing, catalysis and photocatalysis. Interest in anodised tin oxide has only emerged in recent years, Shin [108] among the first to explore this material. Zaraska et al. [109] leveraged the distinctive nanochanneled architecture of anodised films to reduce SnO_2_’s high electron recombination rates. More recently, Lv and colleagues demonstrated the semiconducting advantages of anodised tin oxide in photoelectrochemical water splitting [110].

#### 3.3.1. Niobium

Niobium, as a non-toxic element, is similar to other transition metals such as Ti, Zr and Ta in its ability to spontaneously form an oxide layer on its surface [111,112,113]. Niobium pentoxide (Nb_2_O_5_) is the most stable and chemically resistant form of niobium oxide. Moreover, it exists in several polymorphic forms, all featuring niobium atoms in octahedral coordination [114]. These include B-Nb_2_O_5_ (rutile-like), N-Nb_2_O_5_ and H-Nb_2_O_5_ (both based on ReO_3_-type block structures) [115,116,117]. The most common structures observed in Nb-based nanotubes are orthorhombic or monoclinic, with pore diameters typically ranging from 20 to 50 nm. Over the past few decades, scientists have discovered more than 17 distinct crystalline phases of Nb_2_O_5_, each exhibiting unique structural and functional characteristics. The various polymorphs of Nb_2_O_5_ serve as an ideal model system for exploring structure-property relationships [116].

By carefully selecting the anodisation conditions, such as voltage, processing time and electrolyte composition, it is possible to tailor the morphology of niobium oxide nanotubes, including their diameter, length and wall thickness [114]. Generally, the morphology of niobium synthesised by anodisation is strongly influenced by the electrolyte composition, applied voltage and anodisation time. The mechanism of nanotube formation involves a sequence of five stages, beginning with the presence of a native passive layer of Nb_2_O_5_, followed by pit formation under constant voltage, the growth of these pits into nanopores, the development of the barrier layer and finally the emergence of well-defined nanotubular structures on the niobium surface [112,118]. In electrolytes containing fluoride ions, two competing processes govern the formation of Nb_2_O_5_ nanotube arrays: field-assisted oxidation of niobium and simultaneous chemical dissolution of the oxide layer [119,120].

In acidic electrolytes (e.g., 1 M H_2_SO_4_ + 0.5–2 wt% HF), short anodisation at 20 V for 0.5 h produced nanotubes with diameters of 20–30 nm and lengths of 500 nm [5,121]. Phosphoric acid-based electrolytes (1 M H_3_PO_4_ + 1 wt% HF) enabled the formation of either thin (8–12 nm) and short (180 nm) or wider (90–130 nm) and similarly long (500 nm) nanotubes depending on the applied voltage and duration [122]. In contrast, glycerol-based electrolytes containing NH_4_F facilitated the growth of significantly longer nanotubes (up to 4 μm) with diameters up to 80 nm under 20 V for 2 h [123]. These findings highlight the tunability of nanotube dimensions through precise control of anodisation parameters.

Hexagonal Nb_2_O_5_ films were obtained through annealing in a nitrogen atmosphere and their pseudocapacitive performance was preliminarily evaluated in an organic LiClO_4_ electrolyte.

An innovative method for in situ doping of Nb_2_O_5_ with alkali metal ions (Li^+^, Na^+^, K^+^, Rb^+^ and Cs^+^) was introduced via high-frequency, negative-to-positive pulsed voltage anodisation of niobium foils. At optimal dopant levels and synthesis parameters, the alkali-doped Nb_2_O_5_ electrodes demonstrated a twofold improvement in photoelectrochemical water splitting performance relative to their undoped counterparts, primarily due to enhanced charge carrier concentration and improved interfacial charge transfer kinetics [124].

The oxide films were characterised as amorphous Nb_2_O_5_, which undergoes a phase transformation into an orthorhombic structure upon annealing at 450 °C in air. In another study, the authors selected K_2_HPO_4_/glycerol as the anodisation electrolyte, employing a high-temperature anodisation process for Nb under galvanostatic conditions rather than the conventional potentiostatic approach. This methodological choice influences the oxide film’s morphology, composition and subsequent properties, potentially enhancing its applicability in electronic and optical devices [125]. Their research initiated the evaluation of the capacitive response of these structures to negative potentials in an inert aqueous electrolyte.

Lim et al. employed a versatile synthesis strategy to fabricate core-shell structured T-Nb_2_O_5_@carbon nanocrystals (T-Nb_2_O_5_/C NCs) and TT-Nb_2_O_5_/C composites, aiming to enhance electrochemical performance through structural and conductive optimization [126].

The oxide layer enhances the corrosion resistance of niobium in the human body and is primarily modified through electrochemical methods. Higher oxidation temperatures lead to increased hardness and roughness of niobium surfaces. The enhanced hardness improves wear resistance, reducing material degradation caused by friction. Meanwhile, surface roughness, when optimised within a suitable range, enhances adhesion properties, which can positively influence the cohesion between body tissues and implanted materials. This makes thermally treated niobium coatings highly relevant for biomedical applications, such as implants, where durability and biocompatibility are essential [127]. Generally, its unique characteristics make it a valuable material for numerous technological applications, which are presented in the next paragraph.

#### 3.3.2. Zinc

Anodic zinc oxide (AZO) layers are attracting interdisciplinary research interest. As a commercial process, it was finally launched in the late 1960s, utilising International Lead Zinc Research Organization Inc. (Durham, NC, USA) [128].

Zinc oxide is a wide bandgap II–IV semiconductor material (direct band gap equal 3.37 eV at 300 K) with large free excitation binding energy (60 meV). This material is renowned for its high thermal stability, hexagonal structure and exceptional mechanical strength, making it suitable for demanding applications. However, zinc coatings, despite their widespread use, are prone to staining and rapid tarnishing. They also exhibit susceptibility to atmospheric corrosion, which can compromise their durability over time. Under atmospheric conditions, zinc materials exhibit a certain corrosion resistance in comparison to steel and form a smooth, compact and weather-resistant protective layer. The layer consists of zinc oxide, zinc hydroxide and carbonate, Zn(CO_3_)·Zn(OH)_2_, known as white rust. Addressing these limitations often involves protective treatments, alloying or specialised coatings to enhance their longevity and performance in harsh environments [129,130].

However, electrochemical generation of nanoporous oxide layers on Zn remains still a challenge, even much greater than in cases of other anodic metal oxides. Several strategies leading to the formation of nanoporous or nanotubular ZnO have been already proposed. Nevertheless, anodically formed structures in the majority are sponge-like, granular and completely disordered.

In 1983, Nanto et al. reported that depending on anodising conditions, such as, the electrolyte concentration and applied potential, various zinc oxide films can be formed by anodisation in KOH [131]. The amount of literature on anodising has increased in recent years and allows correlations between the structure formation and the anodising parameters depending on the electrolyte.

Anodic treatment encompasses three primary techniques: electrolytic oxidation, plasma electrolytic oxidation (PEO) and electrolytic etching. Anodisation enhances surface properties through controlled electrochemical reactions. PEO, often referred to as micro-arc oxidation when using DC or iridising when employing AC, produces durable ceramic coatings with advanced functionalities. Meanwhile, electrolytic etching serves as a precision method for modifying surface characteristics, further expanding the applications of anodic treatment processes [132].

Modifying ZnO with metal and non-metal dopants such as Fe, Cu, Ag, Sn, C, N, F, P and S creates localised donor states within its band gap, significantly boosting light absorption and electron mobility. Furthermore, enhancing ZnO photocatalytic efficiency can be achieved by incorporating noble metal deposits on its surface or forming heterostructured photocatalysts with semiconducting metal oxides of differing band gaps, leading to improved charge separation and reactivity [133,134].

Anodisation is an alternative option to increase the corrosion resistance and wear properties of zinc and galvanised layers, as it can generate protective oxide layers [135]. Many types of material have been investigated as surface modification layers for zinc metal anodes for aqueous rechargeable zinc-ion batteries (AZIBs) [136], including carbon based materials [137,138], metal oxides [139,140] organic–inorganic hybrid materials [141], metals and alloys [142,143], metal-organic framework - MOF-based materials [144,145], polymer materials [146], inorganic acid salts [147], metal sulphides [148,149,150] Once coated onto zinc foil, all variants exhibit enhanced electrochemical properties compared to uncoated zinc foil [151,152,153].

Dong et al. explored the formation mechanism of layers on the zinc anode in a 0.1 M NaOH solution, applying voltages between 5 and 12 V for one hour. Their findings revealed two distinct structures: a porous structure emerging around 9 V and a columnar structure forming at approximately 12 V. Separately, Yam et al. investigated the synthesis of ZnO thin films via acidified ethanolic anodising. While this anodising method offers a cost-effective approach for fabricating ZnO nanostructured thin films, its reliance on chemical processes raises environmental concerns. Consequently, there is a pressing need for an alternative fabrication method that minimises chemical usage while maintaining affordability. Notably, the morphology of ZnO thin films is heavily influenced by the applied anodising voltage [154].

ZnO thin films were effectively produced through the anodisation of zinc plates in distilled water at 25 °C with applied voltages ranging from 10 V to 30 V. The structural characteristics of these films were strongly influenced by the anodising voltage. At lower voltages of 10 and 15 V, the films lacked nanoporous features, whereas nanoporous structures emerged at higher voltages of 25 and 30 V. X-ray diffraction analysis confirmed the formation of ZnO with a hexagonal wurtzite crystal structure across all anodised Zn plates within the studied voltage range [155].

Voon et al. and Shetty et al. independently conducted anodisation in deionised water under controlled voltage conditions. Distilled water, with its low electrical conductivity of less than 10 μW^−1^ m^−1^ is generally ineffective as an electrolyte for anodising due to time inefficiencies. Shetty et al. applied voltages between 1 and 9 V over durations ranging from 6 to 12 h, while Voon et al. extended the anodising process to 120 h at voltages between 10 and 30 V. Both studies observed the formation of a nanoporous AZO layer. Additionally, Shetty et al. examined the influence of anodising duration on current density in an aqueous electrolyte (pH 5.8), maintaining a constant voltage of 3 V for up to 12 h [155,156,157].

Potassium hydroxide is commonly utilised as an electrolyte in anodising and plasma electrolytic oxidation of zinc. These processes frequently result in the formation of porous structures, which may take on either a columnar or granular morphology. Such porosity develops when zinc is treated in pure KOH solutions with concentrations ranging from 0.05 to 2 M [128,158,159,160].

Anodisation of hot-dip galvanised steel in a 0.2 M KOH electrolyte successfully produced nanoporous zinc oxide layers. These layers reached thicknesses of up to 500 nm, with current densities ranging from 10 to 50 mA/cm^2^ and anodisation times extending to 600 s. Throughout the process, the voltage remained below 26 V. The resulting AZO layers underwent a noticeable colour shift from beige to brown and exhibited a free surface energy increase of up to 65 mN/m, which is 71% higher than that of pure zinc surfaces [161].

Ono et al. successfully electrochemically synthesised nanoporous ZnO films in a 0.1 M NaOH solution at an applied potential of 10 V. Furthermore, the incorporation of ethylene glycol into the electrolyte enabled the formation of anodic layers with thicknesses reaching up to 90 μm. More recently, our research has led to the successful anodisation of dark nanoporous ZnO films in 1 M NaOH under significantly lower potential conditions [162].

Sreekantan et al. fabricated complex zinc oxide structures in 1 to 6 M NaOH electrolytes. Crystalline zinc oxide in rod and plate-like structure was otherwise observed when zinc nitrate was added to the electrolyte as zinc species or ammonium chloride [163]. In the case of potentiostatic anodisation, the growth mechanism is controlled by the formation of a nanoscale passivation layer (as is also known for aluminium anodisation) on which the zinc oxide nanorods are subsequently grown by field diffusion in a “bottom-up” process [164].

A different category of electrolytes employed in the anodic synthesis of nanoporous ZnO consists of those containing fluoride (F^−^) ions [165]. Shrestha et al. successfully produced hierarchical structures composed of nanotubular ZnO coated with a dense outer layer rich in ZnS [166]. This was achieved through anodisation in an electrolyte solution containing sodium sulphide (Na_2_S) and ammonium fluoride (NH_4_F). In a recent study, Sanz-Marco et al. utilised a comparable electrolyte to fabricate nanostructured ZnO films on a rotating electrode. Their findings indicated that elevating both the anodising voltage and the rotation speed progressively transformed the film’s structure from a nanosponge-like form to a nanotubular architecture [167].

Vanadium ion-doped ZnO nanorods (NRs) were successfully synthesised using an advanced ion implantation technique. Based on XRD and XAS techniques, we confirmed that vanadium dopants were incorporated into the ZnO lattice as V^4+^ and V^5+^ ions. The presence of V^4+^ dopants introduced impurity levels within the forbidden band gap of ZnO NRs, extending their optical absorption into the visible light spectrum. Additionally, the vanadium ion dopants significantly enhanced the charge carrier density in the ZnO NRs. When utilised as a photoanode for solar water splitting, the V-doped ZnO NRs demonstrated a superior photocurrent density of 10.5 μA/cm^2^ at 0.8 V under VIS light illumination, representing a fourfold improvement compared to pure ZnO NRs [168].

The use of appropriate electrolyte and specific anodising conditions can lead to the formation of a great variety of nanostructured ZnO morphologies from nanoparticles, through nanowires, nanoparticles with various morphologies (hexagonal, rectangular, stair), flower-like bunches and nanorods, up to nanoporous layers or even more complex nanoarchitectures, it should be emphasised that very careful optimisation of conditions applied during anodic oxidation is strictly required to achieve the desired morphology of the anodic film [169]. Morphologies of nanostructured zinc oxide have received significant attention owing to their exceptional properties including extremely effective charge carriers transport, high surface-to-volume ratio, enhanced light harvesting and many others [170,171].

As shown above, a great variety of ZnO nanostructures can be formed by simple anodic oxidation of metallic Zn. However, as mentioned before, a slight alteration of anodisation conditions can cause significant changes in the product morphology. Comprehensive studies on the synthesis of nanostructured ZnO films via anodisation in different electrolytes at different voltages, temperatures and over various durations have been performed by Ramirez-Canon and other [172,173].

Careful optimisation of anodising conditions is critical for achieving the desired ZnO nanostructure. The ability to engineer morphologies ranging from nanoparticles [174,175] to nanowires [176,177] and nanoporous layers [178,179] demonstrates the versatility of the anodic oxidation method. While nanoporous semiconductors offer advantages such as high surface area, tunable pore size and ease of functionalisation, electrochemical synthesis of highly ordered ZnO nanostructures remains a challenge.

#### 3.3.3. Copper

Copper is another metal with a strong potential for creating oxide nanostructures through self-organised anodisation. Copper forms two primary oxides: copper(I) oxide (Cu_2_O) and copper(II) oxide (CuO), as well as mixed-phase compounds like the copper-rich Cu_4_O_3_. XPS measurements conclusively determined the oxidation states of copper in its various states [66]. When exposed to moist air, Cu_2_O gradually transforms into CuO. The drive to develop straightforward synthesis methods for copper oxide nanostructures stems from the distinct electronic characteristics of Cu_2_O, CuO and Cu_4_O_3_. CuO, a known p-type semiconductor, exhibits a band gap ranging from 1.2 to 2.16 eV, influenced by factors such as its direct or indirect nature, doping level, morphology and crystallite size. For CuO nanostructures, a reduction in size correlates with a widening of the band gap, typically seen as a blue shift in the optical spectrum. Similarly, Cu_2_O is a p-type semiconductor with a band gap exceeding 2.1 eV. A wide array of synthesis techniques have been developed for producing Cu_2_O and CuO nanostructures, encompassing various chemical approaches such as high-temperature annealing in oxidative environments, sol-gel processing and micelle-assisted precipitation. These methods have enabled the fabrication of diverse nano-structural morphologies—including leaf-like nanocrystals, nanoribbons, nanoflowers, nanorings, nanospheres and multipod architectures—which are well-documented in comprehensive reviews [180,181].

The anodisation mechanism of copper is significantly more intricate compared to that of AAO, due to the simultaneous formation of two distinct copper oxides and the generation of soluble coordination complexes. Even when oxidation is limited to the Cu^+^ state, as examined by Gennero de Chiavlo et al., the process involves a range of complex phenomena [182]. Additional treatments allow oxidising or reducing the formed nanostructure. Copper anodisation also plays a valuable role in renewable energy research, particularly in photocatalytic water splitting for hydrogen and oxygen production. When the photocatalyst consists of a Cu_2_O-CuO composite, it facilitates the generation of oxygen from water under light irradiation. Anodisation of copper in alkaline electrolytes typically results in the formation of copper hydroxide phases, which can subsequently be thermally transformed into CuO or Cu_2_O through annealing [183]. Given the inherent complexity of copper passivation, it presents numerous avenues for in-depth fundamental research. One of the most straightforward electrochemical approaches to passivating a metal—particularly one not yet extensively explored through anodisation—involves using a potentiostat in a three-electrode setup. This allows for potentiostatic experiments within the passivity region, guided by the Pourbaix diagram. Anodisation in aqueous KOH electrolytes typically leads to the formation of nanostructures such as nanoneedles. Stępniowski et al. conducted such a study on copper passivation in a 1 M aqueous KOH solution [184]. Their voltammetric analysis revealed two distinct oxidation peaks, occurring around −450 and −150 mV vs. Ag|AgCl, corresponding to the stepwise oxidation of metallic copper to Cu^+^ and Cu^2+^, respectively.

Pure Cu_2_O films can be synthesised at lower deposition voltages, whereas higher voltages combined with extended deposition times yield metallic copper films. The Cu_2_O films produced exhibit a complex surface morphology and display n-type semiconducting behaviour. Both the structural and compositional characteristics of the films are strongly influenced by the applied deposition voltage [185].

Cu_2_O nanofilms with tunable band structures were fabricated via electrodeposition by altering the chemical environment of the deposition solution, as the band alignment in heterojunctions critically affects device performance and can be tailored through synthesis conditions. By integrating the electrodeposited Cu_2_O films with TiO_2_ nanorod arrays, Cu_2_O/TiO_2_ heterojunctions were successfully constructed [186].

Although most research focuses primarily on pure copper, studies can also be found in the literature that explores the influence of copper on the anodisation of AAO. Vanpaemel et al. conducted a study on the fundamental mechanism for the formation of three-dimensional porous template during the anodisation of Al with less than 1 at% Cu [187]. The copper impurities are pushed downward during the initial phase and accumulate just beneath the planar alumina layer. This enrichment of Cu below the oxide layer is favoured due to its higher free energy of oxide formation compared to that of Al. In a related approach, Kang demonstrated the post-anodisation conversion of WO_3_ to WC by treating the oxide in a CO atmosphere, yielding a cathode material suitable for photoelectrochemical applications [188]. The incorporation of copper impurities into an aluminium film induce the formation of lateral pores that link the vertically oriented porous channels within the AAO structure. A new mechanism has been proposed that connects the observed current density oscillations to the accumulation of Cu at the metal/oxide interface, driven by the cyclic variations in the anode potential. In the other paper [189], authors show 2-D and 3-D AAO membranes formed from pure and Cu doped aluminium films by one-step method at room temperature.

Anodising pure aluminium results in the formation of alumina with uniformly straight, vertically aligned pores, even at room temperature. In contrast, the presence of copper impurities in aluminium films leads to the development of a highly ordered mesh-like structure, characterised by evenly spaced pores in both vertical and horizontal orientations. Mebed attributes the formation of the copper-enriched layer to dislocation movement via the Orowan mechanism, highlighting its role in facilitating the accumulation of Cu [190]. The distributions of Cu in the alloy and the enriched layer on the alloy/film surface were investigated and explained used many experimental techniques. For thin films with an average thickness below 500 nm, Cu atoms tend to migrate toward the surface, where they form patterned precipitates that remain on the film’s exterior. The influence of grain boundary migration on hillock formation in non-passivated thin aluminium films subjected to thermal cycling has also been documented. It has been observed that films undergoing grain growth during thermal stress tend to develop a higher density of hillocks. In other paper, were fabricated from Cu-doped aluminium films deposited on p-type silicon wafers by anodisation and obtained results were similar [99].

Nanowires produced via single-layer porous anodic alumina templates typically exhibit limited mechanical strength. To enhance structural integrity, multilayer AAO templates were employed for the fabrication of Cu and Ni nanowires using pulse electrochemical deposition (PECD). The anodisation process was conducted on Al-0.5 wt% Cu/Al-1 wt% Si and Al-0.5 wt% Cu/Al-1 wt% Si/Al-0.5 wt% Cu multilayer films in H_2_SO_4_ and C_2_H_2_O_4_ electrolytes, resulting in templates with varied morphologies. The resulting nanowires showed improved mechanical robustness and unique structural characteristics, including vertically aligned top-layer wires with lateral bridging and lower-layer wires forming shrub-like structures, with diameters between 50 and 70 nm [191].

As presented in paper [191] the nanowires exhibit uniform spatial distribution and diameters closely matching the pore dimensions of the original template. Furthermore, the presence of lateral interconnections between nanowires contributes significantly to the mechanical reinforcement of the vertically aligned structures. Moreover, Abd-Elnaiemet et al. [191] prepared porous AAO thin films with interconnected pores through Cu-doped aluminium films deposited on p-type silicon wafers and found that the oxide film evolution and growth mode drastically changed with the anodisation parameters (i.e., anodisation voltage and time). Other teams presented gas diffusion electrodes with adjustable pore morphology [192]. These investigations revealed that the deposition of a compact Cu layer on the membrane surface effectively blocks the underlying pore openings, thus limiting excessive electrolyte infiltration into the pore channels responsible for gaseous CO_2_ transport. In contrast, the incorporation of Cu within the internal pore architecture of the newly developed membrane electrodes significantly enhanced C_2_H_4_/CO selectivity—by up to a factor of three—compared to configurations where Cu was exclusively deposited as a dense surface layer. Moreover, it was observed that, even after electrolyte wetting, macropores allowed continued access of gaseous CO_2_ to the embedded Cu sites, whereas CO_2_ reduction reaction (CO_2_RR) was entirely suppressed within wetted nanopores, underscoring the critical influence of pore dimensions on catalytic performance.

Ma et al. employed the polyol synthesis technique to deposit Cu_2_O films onto copper substrates, marking the first reported fabrication of Cu_2_O films composed of aligned two-dimensional single-crystal nanosheets [193]. Subsequently, various substrates, including fluorine-doped tin oxide glass, porous nickel meshes, TiO_2_ nanotube arrays and others, have been utilised for the growth of copper oxide nanostructures [194,195,196].

In the article [197], copper was incorporated onto the surface of TiO_2_ nanotube layers by alternating current electrodeposition after anodisation of a titanium alloy. The electrodeposited copper particles, present both on and within the nanotubes, underwent a transformation into Cu_3_(PO_4_)_2_·3H_2_O microflowers after immersion in a phosphate buffer solution. The antibacterial activity of the copper-modified surfaces was influenced by the morphology of the deposited copper and its ability to release Cu^2+^ ions.

A copper nanopillar array (CuNPA)-filled AAO film was successfully synthesised via the pulse electrodeposition technique for application in electronic interconnections [198]. During high-frequency pulse deposition, Cu ions effectively diffused into the AAO nanopores during the off-time, while the on-time enabled the uniform filling of these pores with CuNPA. Within the composite film, the AAO matrix served as a dielectric and thermal insulator, while the embedded CuNPA facilitated high-density electrical conduction through nanoscale channels. The resulting film exhibited enhanced thermal conductivity compared to conventional conductive adhesives. Furthermore, the film demonstrated strong solderability with copper substrates, confirming its compatibility with advanced packaging technologies for high-density and power electronic devices.

Additionally, a Cu_3_(PO_4_)_2_/PAA-g-PVDF (polyvinylidene fluoride) composite membrane was fabricated by alternately immersing the PAA-g-PVDF substrate in CuSO_4_ and phosphate buffer solutions [199,200,201]. The resulting Cu_3_(PO_4_)_2_ nanoparticles were uniformly embedded within the membrane’s porous structure. This mineralised membrane exhibited excellent anti-fouling performance when applied to high-viscosity crude oil-in-water emulsions.

Generally, incorporation of Co into the pores of AAO has proven to be an effective method to create functional coatings with a variety of practical uses. AAO layers modified with copper [198,202,203,204,205] or a combination with nickel [204,206,207] have been also utilised in many applications. The advantageous characteristics of Cu/Ni-modified AAO coatings can be further enhanced by introducing additional elements like iron and cobalt [208,209,210,211].

Latest study investigates how extended electrodeposition time influences the dielectric properties of Cu/AAO nanocomposites. Copper nanowires were deposited into the pores of AAO templates prepared via a two-step anodisation process. The dielectric characteristics—specifically, the real and imaginary components of the effective complex relative permittivity and the effective AC conductivity—were evaluated for both bare AAO templates and Cu/AAO nanocomposites. The findings reveal that prolonged electrodeposition enhances both permittivity and AC conductivity, underscoring the potential of metal-filled AAO structures for tunable dielectric performance in advanced capacitor technologies [212].

Many recent studies employ numerical methods, computer simulations and ab initio calculations to model and design the properties of novel nanomaterials. The simulation study [213] examined the thermal and structural characteristics of an aluminium piston subjected to anodisation with a copper alloy. Within the Al-Cu composite oxide layer, copper was found to form a network structure with oxygen ions. The anodisation process was influenced by the morphology and dimensions of the copper particles. Mechanical alterations to the casting surface were observed to affect the anodisation behaviour. Copper particles embedded in the anodic film contributed to improved wear resistance; however, the use of coarser-textured copper particles resulted in reduced wear resistance of the anodic layer. The findings indicated that the copper-anodised aluminium piston exhibited enhanced structural rigidity and experienced lower strain and deformation compared to its uncoated counterpart. Another study focused on both experimental and theoretical investigations into how natural convection near nanoporous anodic aluminium oxide templates affects nanowire deposition. Copper nanowires were electrochemically deposited using vertically oriented templates aligned with the direction of gravitational acceleration under varying overpotentials. To describe the process, a deposition model was formulated incorporating the Navier–Stokes equations and ionic diffusion dynamics [214].

#### 3.3.4. Magnesium

Magnesium is a lightweight structural metal known for its high strength-to-weight ratio, excellent machinability and abundance, making it an attractive material for applications in aerospace, automotive and electronics industries. However, its widespread use is significantly limited by one major drawback—poor corrosion resistance, especially in humid or saline environments. Magnesium corrodes in aqueous environments primarily through an electrochemical reaction with water, forming magnesium hydroxide and releasing hydrogen gas. To overcome this limitation, surface treatments, such as galvanising anodisation or even PVD and CVD techniques, are employed, forming a protective oxide layer that enhances the corrosion resistance of magnesium and its alloys while often improving surface hardness and appearance. After banning chromium-based coatings in many industrial areas, plasma electrolytic oxidation (PEO) became the most popular industrial method for enhancing magnesium alloys.

Anodisation has the advantage over the other methods because the formed layer is not only stable in corrosive environment but also non-conductive, scratch-resistant and has a relatively rough morphology which allows for easier deposition of a different coating on top of it. It also scales up well and thus it was implemented as a standard industrial method. The anodisation of magnesium and its alloys is more complicated in comparison to aluminium. Although magnesium is more reactive than aluminium, its porous oxide formed during anodisation is less stable and dissolves in many electrolytes. Consequently, the resulting anodic oxide layer is porous, rough and lacks the protective quality seen in aluminium oxide coatings. This is due to the low Pilling–Bedworth ratio of MgO (0.81), indicating insufficient surface coverage.

In order to sustain the oxide growth, higher voltage is required while Al readily forms stable, dense coating with the barrier layer at relatively low voltages in acidic electrolytes. The anticorrosion property can be improved by suppressing the anodic or cathodic reactions. The higher voltage of magnesium anodisation causes micro-arc discharging making the process similar to plasma electrolytic oxidation rather than conventional anodisation. The anodised layer start to form at lower voltages or current density but the film changes characteristics during the process and breaks down at higher voltages [215]. Further formulation of the anodised layer at high voltages is thus breaking down and repairing of the film formed at low voltages accompanied by sparking. This phenomenon occurs when the voltage reaches a critical value (the breakdown voltage) at which the oxide layer loses its insulating properties and starts to conduct electricity. The discharges are reported to change size and travel on the surface of a sample resulting in fluctuations in current and potential [216].

In alkaline conditions, magnesium alloys develop a passive Mg(OH)_2_ film on the surface. Nevertheless, both MgO and Mg(OH)_2_ are only slightly soluble in water, offering limited long-term protection. The presence of aggressive ions such as chloride, bromide or sulphate disrupts these films, accelerating corrosion. Enhancing corrosion resistance can be achieved by inhibiting either anodic or cathodic reactions.

A range of surface treatment methods are available to improve the performance of magnesium, including increasing wear resistance, corrosion protection, hardness and achieving a decorative finish. Dry surface treatments are typically more environmentally sustainable and can deliver precise, aesthetically pleasing coatings. On the other hand, wet methods are cost-effective, user-friendly and provide diverse functional surface enhancements. Notably, Shashikala et al. evaluated the corrosion resistance of several chemical conversion coatings—such as cerium oxide, stannate, chromate and galvanic black anodising—applied to AZ31B magnesium alloy [217]. Song et al. studied the anodising behaviour of AZ31 magnesium alloy in a NaOH solution containing boric acid, sodium tetraborate and various additives. Their findings showed that as anodising time increased, the initially formed oxide film developed into a thicker structure with a porous outer layer and a denser, more compact inner layer directly adhering to the magnesium alloy substrate [218].

#### 3.3.5. Tantalum

This chapter examines the synthesis and recent advances in fabricating micro- and nanostructured anodic tantalum oxides (ATO). Tantalum oxide (Ta_2_O_5_) is valued for its high melting point, high transparency and remarkable refractive index approximately 2.2 at 633 nm, making it a vital material for a range of technological applications [219,220,221,222]. Tantalum can exist in different oxidation states—forming Ta_2_O_5_ with tantalum in the +5 state or TaO_2_ with it in the +4 state. The Ta_2_O_5_ phase is thermodynamically the most stable [223,224]. Moreover, at the nanoscale, Ta_2_O_5_ exhibits exceptional electrical, optical and chemical stabilities. Typically, high-purity tantalum (approximately 99.9%) is selected as the starting material and is commonly available in forms such as foils, sheets, discs or rods [225,226,227,228,229,230,231]. Nevertheless, controlling the anodisation process to reproducibly form stable nanoporous or nanotubular structures remains challenging.

Nanostructured Ta_2_O_5_ films are typically produced by anodisation—a cost-effective, scalable electrochemical method. In this process, an electric potential is applied between a tantalum metal substrate (serving as the anode) and a counter electrode (often platinum, lead or graphite), thereby inducing charge carrier transfer and forming a protective oxide layer with controllable thickness [36,198,232]. With careful adjustment of anodisation parameters, the surface morphology can be tuned to yield either dense, compact films or highly regular porous architectures—ranging from nanotubular to multilayered structures—often accompanied by deliberate chemical modifications.

Historical work has laid the groundwork for our modern understanding of ATO formation. The pioneering studies of Vermilyea in the 1950s [233], followed by investigations from Gunzel in 1972 [234], F. Arifuku [235] in 1978 and Shimizu in 1996 [236], expanded our understanding of not only the growth kinetics of tantalum oxide but also the incorporation of electrolyte species (such as sulphur and phosphorus from H_2_SO_4_ and H_3_PO_4_) into the oxide matrix. Most authors employed an anodisation process in a 0.5 M H_2_SO_4_ electrolyte to fabricate Ta_2_O_5_ films. Albella et al. [237] later confirmed that phosphorus was incorporated into the oxide as ${\mathrm{PO}}_{4}^{3-}$ anions, while further studies by Shimizu’s group [236] probed the mobility of various cations and anions within the film. In 2005, Sieber et al. [230,238] were the first to report the formation of porous ATO layers on Ta foil using a 1 M H_2_SO_4_ (sulphuric acid) solution with a minor hydrofluoric acid HF content (0.1–3 wt%); they observed oxide thicknesses on the order of 130 nm. Subsequent research underscored the critical role of the H_2_SO_4_/HF ratio in achieving well-ordered nanoporous or nanotubular architectures [125,230,239,240]. From released of these papers, the vast majority of research on Ta anodisation has exploited electrolytes based on mixtures of HF and H_2_SO_4_ to produce nanoporous surfaces or membranes [241,242,243,244]. From released of this paper, the vast majority of research on Ta anodisation has exploited electrolytes based on mixtures of HF and H_2_SO_4_ to produce nanoporous surfaces or membranes.

Anodic oxidation in electrolytes can induce a controlled porosification of tantalum, where both acid concentration and the duration of application critically determine the pore architecture. Higher electrolyte concentrations boost the current density and promote the formation of a uniform, porous oxide; however, the process tends to be associated with unstable electrochemical conditions [245]. Alternatively, researchers have also explored fabricating nanostructured tantalum oxide layers in a non-aqueous, acid-free electrolyte composed solely of ethylene glycol and water. The next step of investigations involved improving this method by introducing additives such dimethyl sulphoxide (DMSO) [225,246,247]. Early on, Allam et al. spearheaded investigations into the influence of additives—including EG, DMSO, H_3_PO_4_, —in aqueous H_2_SO_4_/HF mixtures [248]. Their work demonstrated that these additives could drive the vertical alignment of Ta_2_O_5_ nanotube arrays. In parallel, investigations in nonaqueous media have shed light on alternative strategies. For instance, Wei et al. explored anodisation in a glycerol-based electrolyte containing small amounts of ammonium fluoride (NH_4_F), showing that the applied potential, fluoride concentration and water content critically influence the porosity [242]. Moreover, Momeni et al. found that while a pure glycerol/NH_4_F electrolyte yields irregular films, a combined glycerol–EG mixture with NH_4_F produces highly ordered nanoporous surfaces [243]. Yu et al. even reported a novel coral-like nanoporous morphology using a mixture of EG, H_3_PO_4_, NH_4_F and water, while uniformly ordered nanoporous films have also been obtained in fluoride-free electrolytes composed of glycerol and dipotassium phosphate operated at elevated temperatures [249].

Another way to achieve desired morphology is increasing the anodisation voltage; for example, porous hafnium oxide films grew from approximately 8 μm at 10 V to 18 μm at 60 V, underlining the feasibility of fabricating self-organised, template-free HfO_2_ nanotube layers [230,250].

Moving on to the details, employing a mixture of H_2_SO_4_ and HF lead to the formation of a highly ordered nanoporous layer [240,248,251,252,253].

El-Sayed et al. [253] found that applying low anodisation potentials (10–20 V, well below the Ta_x_O breakdown potential of approximately 200 V) for durations ranging from 30 s to 10 min in concentrated H_2_SO_4_/HF mixtures (16.4 and 2.9 M, respectively) can yield exceptionally ordered nanoporous structures. Beyond the formation of nanostructures, the corrosion resistance of anodically formed Ta_2_O_5_ has been a central focus. Hashimoto et al. [254] showed that forming a dual-layer of Ta_2_O_5_ and MoO_2_ on Ta–Mo alloys bolsters the diffusion barrier and improves corrosion protection. In studies on Ta–Nb alloys, Robin [255] observed that increasing both the H_2_SO_4_ concentration and the temperature escalates the corrosion rate—although some researchers argue that prolonged exposure to harsh conditions may promote high-temperature creep and elemental segregation, thereby heightening the risk of galvanic or selective corrosion. Surface modification via anodisation has emerged as an efficient and environmentally benign method to enhance Ta’s corrosion resistance, given its ease of control and low cost. For example, Vermilyea et al. [233] produced Ta oxide films in H_2_SO_4_ solutions and showed that variations in anodisation voltage and duration can profoundly change the film’s evolution, while Sieber et al. [230] documented that the anodic oxide on Ta typically grows to about 100 nm–increasing its protection by far more than the natural passive layer.

A key feature of anodic tantalum oxide films is their dual-layer structure: porous external layer overlays a dense inner barrier that is paramount for corrosion resistance [256]. As the thickness of the anodic film increases, unstable low-valence tantalum species (Ta^2+^ and Ta^3+^) gradually convert into the stable Ta^5+^ state [257]. Even minor adjustments in anodisation parameters—such as voltage and duration—profoundly impact the nucleation and growth kinetics and thus the final corrosion properties. Despite substantial experimental progress, the comprehensive mechanism behind the evolution and corrosion behaviour of these films on tantalum remains incompletely understood [258].

The anodic fabrication of Ta_2_O_5_ has been systematically explored in diverse media—including inorganic solutions (H_2_SO_4_, H_3_PO_4_, NH_4_F, Na_2_SO_4_), organic solvents (oxalic acid, glycerol, EG) and their mixtures (for instance, H_2_SO_4_ + EG + NH_4_F)—over a broad range of potentials (typically from 10 to 200 V). These conditions give rise to ATO layers with structures varying from zero-dimensional isolated particles to fully three-dimensional networks [259]. Precise control over nanotube dimensions has also been achieved. Horwood et al. [260] demonstrated that the nanotube length of Ta_2_O_5_ could be tuned from 50 up to 1000 nm by varying HF concentration and the anodisation time. Complementary work by Wei et al. [242] showed that nanoporous layers formed in glycerol/NH_4_F systems, while Xia [261] and Fialho et al. [245] reported similar morphologies using EG-based electrolytes at potentials around 15 and 40–60 V, respectively. In contrast, studies using one-component electrolytes (e.g., Na_2_SO_4_, H_2_SO_4_ or H_3_PO_4_) revealed that anodisation parameters had a limited effect on the morphology. Finally, investigations by Wen et al. [262] under constant current conditions in concentrated H_2_SO_4_/HF mixtures revealed that increasing the anodising current from 75 to 150 mA/cm^2^ promotes greater nanotube length and diameter. Only a handful of studies have turned to acid-free systems, which makes the further exploration of such environments particularly intriguing.

#### 3.3.6. Iron

Fe_2_O_3_ has noteworthy applications due to its diverse polymorphs, including hematite (*α*-Fe_2_O_3_) and maghemite (*γ*-Fe_2_O_3_). Iron can adopt multiple oxidation states, with Fe_2_O_3_ being the most prevalent. Among these, hematite stands out as the most chemically and thermodynamically stable phase; it is abundant and non-toxic in nature, exhibits photocatalytic properties, and is proposed as a promising electrode candidate in lithium-ion batteries. Additionally, it can be synthesised and structured into nanopores and nanotubes, substantially enhancing its surface-to-volume ratio. These properties, in addition to its widespread availability and non-toxicity, make haematite a material of interest for photocatalysis. Moreover, it can be produced and nanostructured into nanopores and nanotubes, greatly increasing its surface-to-volume ratio. At 2.2 eV, the bandgap of haematite is lower than those of materials commonly used in photocatalysis, such as titanium (±3.2 eV).

Nanoporous iron oxide was initially synthesised by Prakasam in 2006 [83]. From this paper porous iron oxide nanostructures have garnered significant attention due to their diverse oxidation states, structural versatility and wide-ranging applications. These materials can be synthesised through multiple techniques, including soft and hard templating approaches [263]. Smarsly et al. demonstrated the fabrication of crack-free mesoporous *α*-Fe_2_O_3_ and *α*-FeOOH thin films via the evaporation-induced self-assembly method, employing a diblock copolymer poly(isobutylene)-block-poly(ethylene oxide) (PIB-b-PEO) as a template [264]. Similarly, Ijao et al. successfully synthesised ordered mesoporous (OM) Fe_3_O_4_ with crystalline walls by reducing OM *α*-Fe_2_O_3_ (corundum structure) to Fe_3_O_4_ spinel, followed by oxidation to *γ*-Fe_2_O_3_ [265].

Porous iron oxides possess exceptional functional properties that render them highly effective for a range of advanced technological applications. Their performance is further enhanced by controlled synthesis methods and precisely engineered structural features, which significantly bolster their utility in fields such as energy storage, biomedicine and environmental remediation. Specifically, nanotubular and nanoporous forms of hematite have shown superior performance as photoanodes in water splitting, photoelectrocatalysis and photoelectrochemical hydrogen or oxygen generation. The nanostructuring helps to prevent the recombination of photogenerated charge carriers, thereby improving efficiency. Beyond energy applications, hematite nanostructures have also been employed in gas sensing technologies. Fabrication of these nano iron oxide films has been achieved using ethylene glycol-based electrolytes containing varying concentrations of ammonium fluoride (NH_4_F) and water (H_2_O). In related research, other authors have developed highly ordered metal oxides of several transition metals—including tungsten, iron, cobalt and niobium—using anodisation techniques combined with pretexturing processes. This method has proven effective in producing well-organised porous oxide structures, such as anodic porous alumina and, more recently, anodic porous titania, both demonstrating the benefits of precise structural ordering for enhanced material performance [266].

Yu et al. demonstrated a two-step strategy to synthesise Fe_2_O_3_ hollow nanorods grafted onto TiO_2_ nanotube arrays (TNAs). First, FeOOH nanospindles were uniformly deposited on both inner and outer surfaces of the TNAs through hydrolysis of Fe^3+^ ions. Subsequently, thermal treatment converted FeOOH into Fe_2_O_3_, forming hollow nanorods integrated into the TNAs (TNAs@Fe_2_O_3_) [267]

In a separate study, Del Olmo provides a comprehensive review of anodic oxidation techniques for fabricating nanostructured oxide films on Fe-based alloys and steels. This work systematically examines how various anodisation parameters—including alloy composition, electrolyte chemistry, voltage/current conditions and post-treatment steps such as annealing—affect the structural, compositional and functional properties of the resulting oxide layers. The review also highlights a wide range of application areas for these anodically formed films and offers valuable insights into optimizing process conditions to tailor oxide properties for specific technological uses [26].

Pinilla et al. described an alternative synthetic route for the preparation of highly ordered metal membranes containing Fe and Zr in different layers [268]. In this paper, two different membrane configurations of bimetallic membranes were obtained—Zr (40 nm)/Fe (40 nm)/AAO and Fe (40 nm)/Zr (500 nm)/AAO—by ion-beam sputtering deposition. The researchers concluded that the varying sticking parameters of individual metals significantly affect how pores are filled within the ordered structure. This observed effect provides a basis for optimising the morphology of the investigated multimetallic membranes, as well as comparable systems.

The first studies of the Marquez group were concentrated on the effective fabrication of highly ordered nanoporous membranes using a one-step synthesis approach. Later investigations involved the deposition of various metals onto AAO membranes via ion-beam sputtering. This method enabled the production of membranes composed of individual metals, such as Zr or Fe, as well as multilayered structures, including configurations such as Hf/Zr/AAO and Nb/Zr/AAO [268,269,270,271,272]. Huang et al. demonstrated that employing bi-metallic Zr–Fe films as catalysts enhances both the length of carbon nanotubes and their field emission performance, in comparison to ones synthesised with only iron catalysts [273].

In a study conducted by Jinwei Cao et al., [274], anodised iron oxide films exhibiting a micro–nano structured morphology were synthesised through a combination of heat treatment and anodisation. The precise adjustment of these oxide films at the micron scale was accomplished by modifying the spatial distribution of cementite and ferrite on the substrate via thermal processing. Annealing at 300 °C was identified as an optimal condition for enhancing the crystallinity of iron oxide while preserving its intricate micro–nano architecture. Although higher annealing temperatures further improved crystallization quality, they also induced the collapse of nanoporous structures, leading to significant alterations in surface topography.

The iron oxide films formed on annealed substrates demonstrated the highest specific capacitance among the examined samples, reaching 35.3 mF/cm^2^ at a scan rate of 20 mV/s. It was observed that the positional arrangement and dimensions of cementite and ferrite on the substrate were effectively controlled through thermal treatment. Subsequent anodisation resulted in the development of nanoporous structures at the ferrite sites and micron-scale dimples at the cementite locations. The as-anodised iron oxides exhibited an amorphous phase, while post-annealing treatment facilitated crystallization. Notably, annealing at 300 °C preserved the integrity of the original morphology, whereas elevated temperatures induced the disintegration of structural features and a reconfiguration of surface characteristics [274].

In a study conducted by Zhang and Wang, micro–nano structures were successfully engineered on Al-Li alloy through controlled hydrothermal treatment, with sodium formate concentration and hydrothermal temperature serving as key parameters. The research investigated the directional growth and morphological characteristics of these structures, revealing distinct surface patterns influenced by temperature variations [275]. At hydrothermal temperatures of 110 and 130 °C, the resulting micro–nano structures exhibited overlapping leaf-like nano-plates, with nordstrandite as the predominant phase. However, at 150 °C, the structures transitioned into flower-like formations, where aluminium oxide hydroxide became the dominant compound. The fabricated micro–nano structures consisted of a crystalline mixture of aluminium oxide hydroxide and aluminium hydroxide (Al(OH)_3_), displaying well-defined morphological features. This structural evolution corresponded to a morphological shift, where leaf-like configurations gradually transformed into flower-like architectures. These findings underscore the significance of hydrothermal processing in tailoring the micro–nano surface features of Al-Li alloys for potential functional applications [275].

It is imperative to develop smooth, nanoporous iron oxide films with minimal defects on plain carbon steel (CS) to enhance its photocathodic protection in corrosive environments. To achieve this, a high-temperature annealing treatment was employed to modify the metallographic structure of CS. Specifically, the steel was heat-treated in a muffle furnace at 900 °C for 30 min, a process designed to reconfigure the microstructure prior to subsequent processing. Following annealing, the CS underwent anodisation in an aqueous solution of ammonium fluoride and ethylene glycol, leading to the formation of a uniform nanoporous oxide film. This film was then calcinated at 450 °C for 4 h under a nitrogen atmosphere to transform the as-anodised layer into a crystalline iron oxide film. The sequence of these thermal treatments was critical for controlling the substrate’s microstructure and ensuring the successful fabrication of the desired film.

The high-temperature annealing converted the initially elongated ferrite grains, which varied widely between 20 and 400 μm, into uniformly equiaxed grains with an average size of approximately 60 μm. This transformation resulted in a reduced density of grain boundaries, thereby lowering the surface reactivity and promoting the formation of a smoother nanoporous iron oxide film. Additionally, the diminished presence and size of pearlite precipitates contributed to a reduction in defects such as dimples, while the alleviation of grain strain helped limit crack formation. Consequently, the smooth nanoporous iron oxide film exhibited lower charge transfer resistance, an order of magnitude higher donor density and improved anodic and cathodic reaction activities through the generation of more photoinduced electrons compared to films deposited on untreated CS [276].

Another study focused on investigating the formation of nanoporous iron oxide layers under varying anodisation conditions. Three key parameters were examined: anodising temperature, the application of a magnetic field and electrolyte stirring. The results demonstrated that electrochemical anodisation of iron in an ethylene glycol-based electrolyte containing water and fluoride ions effectively produces nanoporous iron oxide layers. anodising at 10–20 °C was determined to be optimal for establishing the desired nanoporous architecture, while higher temperatures led to the structural degradation of the nanoporous layer. Furthermore, the formation of nanopore arrays was achieved under both static conditions without an applied magnetic field and dynamic conditions with a magnetic field, underscoring the process’s adaptability. Although the as-formed anodic layers were initially amorphous, subsequent heat treatment in air enabled their conversion into crystalline phases, specifically hematite and magnetite. X-ray diffraction analysis confirmed that annealing at 500 °C resulted exclusively in the presence of hematite and magnetite and X-ray photoelectron spectroscopy revealed that the annealing process significantly reduced the fluoride ion content within the anodic layers [277].

In alloy systems, a simple yet effective approach has been developed to construct bionic superhydrophobic surfaces characterised by micro/nanostructured topographies that significantly boost corrosion resistance. This technique was applied to an iron–carbon alloy substrate through a combination of one-step anodic oxidation and a precisely controlled annealing process, resulting in the formation of a protective “armour-like” layer. The study underscored the importance of the alloy’s inherent metallographic properties and their retention during oxidation as essential factors in forming such bionic surfaces. The method involves strategically modifying the anodised oxide layer via thermal treatment, which not only shifts the wetting behaviour but also converts the surface from hydrophilic to superhydrophobic. Notably, annealing at 500 °C transformed the original iron oxide nanosheets into a dense, micro/nanostructured resistive film. This transformation, coupled with the application of a low-surface-energy coating, enables the surface to trap a stable interfacial air layer, serving as an effective barrier against corrosive agents. Consequently, the engineered surface achieves outstanding superhydrophobicity with a contact angle of 155.43° and exhibits markedly enhanced corrosion resistance, evidenced by a two-order-of-magnitude decrease in corrosion current density [278].

Prakasam reported a method for synthesizing self-organised nanoporous hematite through potentiostatic anodisation of iron foil, offering precise control over pore dimensions by adjusting electrochemical conditions. By varying the applied voltage and electrolyte composition, nanopores with diameters in the 50–250 nm range and lengths between 300 and 600 nm were produced. The resulting thin pore walls facilitate efficient transport of photogenerated holes to the electrolyte, but electron mobility toward the underlying iron substrate is impeded by recombination losses, primarily due to the increased barrier layer thickness and suboptimal crystallinity of the oxide [83].

## 4. Applications of Functionalised Anodised Matrices

### 4.1. Energy Applications

In recent years, nanostructures obtained after being anodised have garnered significant attention, owing to their unique properties and wide-ranging potential applications in energy systems. AAO can find valuable applications in many energy storage and conversion systems: Electrochemical Energy Storage Systems, including electrochemical capacitors, batteries and fuel cells. Due to the intermittent nature of renewable energy sources, there is a critical need for efficient energy storage systems capable of storing and delivering energy quickly and reliably. Alternative strategy involves harnessing mechanical energy and transforming it into electrical energy using piezoelectric nanogenerators, which exploit the piezoelectric effect for efficient energy harvesting. These electric systems are being explored alongside other storage technologies such as pumped hydro storage, thermal energy storage, compressed air energy storage and flywheel storage.

Anodisation has emerged as a particularly promising method for synthesizing nanosize layers and materials, offering precise control over composition, morphology, physicochemical and mechanical properties. The electrodeposition of binary alloy nanostructures within porous templates (e.g., AAO) provides a highly adaptable platform for engineering both structural and physicochemical characteristics. By systematically tuning deposition parameters and applying appropriate post-deposition treatments, the electric performance of these nanostructures can be effectively tailored to meet specific functional requirements. These way go to the improvement in electrical conductivity of the energy devices.

In 1995, Masuda and Fukuda introduced the two-step anodisation method for fabricating well-ordered AAO, sparking ongoing efforts by researchers to refine and enhance their production process [54].

Nanostructured niobium oxides nanotubes have been intensively studied for their use as high-performance anodes in lithium-ion batteries. However, far less attention has been paid to their use as a negative electrode in supercapacitors, which is a relatively new research direction. Groundbreaking in this context was the work of Upadhyay, who first obtained nano-Nb_2_O_5_ channels by high-temperature anodisation [279]. These attributes make them particularly well-suited for use in electronics and energy storage systems—where performance, efficiency and longevity are paramount. Anodised films can be used to create dielectric layers in electrolytic capacitors, which are essential for energy storage. Anodised Nb_2_O_5_-based materials are readily applicable as supercapacitor electrodes without requiring further processing [125]. Nb_2_O_5_ materials have been extensively investigated across diverse fields, including electrochemistry, lithium-ion batteries and catalysis. Other range from photoelectric devices, ionic liquid gating, microelectronic catalysis, gas sensors, high-performance oxide glasses and electrochromic devices, to emerging applications in photocatalysis, solar cells, optoelectronics, batteries and photodetectors. Integrating Nb_2_O_5_ with conductive carbon materials to mitigate its intrinsic low conductivity and improve charge transfer has gained increasing attention for supercapacitor electrode applications. Notably, hybrid materials like T-Nb_2_O_5_/graphene and Nb_2_O_5_/carbon nanotubes have shown impressive power densities and robust cycling performance [280,281]. This breakthrough not only enhances material uniformity but also establishes a compelling foundation for advancing lithium-sulphur battery technology. By optimizing the positive electrode composition, this method paves the way for higher capacity energy storage solutions, reinforcing its potential as a transformative approach in next-generation battery development [282].

As mentioned above, iron oxide polymorphisms (FeO, Fe_2_O_3_ and Fe_3_O_4_) have attracted intense research interest in, for example, lithium-ion batteries [283], supercapacitors [283] and many other, because of their photoelectrochemical and electrochemical properties, eco-friendliness, earth abundance and low cost.

Among various transition metal oxides, Fe_2_O_3_ stands out as a highly promising material for energy storage due to its high theoretical capacity (≈1005 mAh/g), natural abundance, low toxicity and cost-effective processing. To address the challenges associated with large volume changes during lithiation, which can degrade performance, hollow or nanotubular structures are employed to buffer these mechanical stresses. Additionally, structural modifications of Fe_2_O_3_ nanotubes further enhance their electrochemical behaviour.

Building on earlier studies involving metals with hexagonal close-packed (hcp) lattices, similar anodisation experiments were carried out on three body-centred cubic (bcc) metals: niobium, tantalum and iron. The results aligned with prior findings, emphasizing the influence of crystallographic structure on the resulting oxide morphology. Specifically, the study revealed a strong correlation between the metal’s lattice structure and the formation mechanism of anodised films. In face-centred cubic (fcc) metals, anodisation typically yields highly ordered nanoporous films with pores arranged in a hexagonal close-packed fashion. In contrast, hcp metals predominantly form nanotubular structures, while bcc metals tend to develop disordered porous films. These findings reinforce the idea that the complexity and packing density of a metal’s crystal lattice dictate not only whether the oxide layer adopts a nanoporous or nanotubular architecture, but also the degree of ordering within the resulting structure. Generally, metals with simpler lattice geometries (fcc and bcc) produce nanoporous morphologies, whereas more complex lattices (hcp) favour nanotube formation. This intrinsic link between crystallographic structure and anodisation behaviour underscores the fundamental role of metallographic features in directing the microstructural evolution of anodised metal oxides [284,285]. One of the application of TiO_2_ is dye-sensitised solar cells (DSSCs), whose construction is based on a thick layer of TiO_2_ with dye adsorbed on the surface of this layer to form an anode. An electrolyte containing redox vapour provides the contact between the anode and cathode. The dyes are designed to absorb radiation from the visible range while being stable in the presence of radiation and having groupings that allow them to be permanently bound to the semiconductor surface. Studies have shown that the use of TiO_2_ nanotubes in DSSCs enables unidirectional charge transport and reduces charge carrier recombination, which significantly increases the performance of TiO_2_ nanotube-based DSSCs compared to cells built with other nanostructured forms of TiO_2_ [286]. Using TiO_2_ nanotubes, an increased electron diffusion path length can be achieved than in TiO_2_ nanoparticles [3]. In addition, the deposition of an additional layer of TiO_2_ nanotubes on TiO_2_ nanotubes has been shown to further improve DSSC performance. For pure layers, the record yield is 5.2% and for a mixture with TiO_2_ nanoparticles, it is 7%. TiO_2_ nanotubes synthesised by anodic oxidation have also been used in lithium-ion batteries as a replacement for thin films obtained from TiO_2_ powder. Fang et al. 2009 [287] were the first to use anodic TiO_2_ nanotubes in lithium-ion batteries. For this purpose, layers of TiO_2_ nanotubes in anatase crystalline form were obtained in an electrolyte containing glycerol, water and ammonium fluoride. The resulting material with an outer diameter of 50–60 nm, a length of 1 μm and a wall thickness of 10–15 nm showed a total capacity of 90 mAh/g. Further work showed that the use of TiO_2_ nanotubes in the amorphous form provided more than twice the capacity (229 mAh/g) of TiO_2_ nanotubes in anatase form (108 mAh/g). This result was consistent with later reports by Ortiz et al. [288], who obtained anodic layers of TiO_2_ nanotubes on Si substrates sputtered with metallic titanium

#### 4.1.1. Battery Components

Typical energy devices consist of three fundamental components: an anode, a cathode and an electrolyte. The anode is the negative electrode where oxidation is present, while the cathode is the positive electrode where reduction occurs. The electrolyte is a material that conducts electricity through the movement of ions (charged particles). In a battery, it’s the medium that allows ions to travel from the anode to the cathode. Their surfaces are specifically engineered to maximise ion and electron conductivity, increase current densities and enhance overall device efficiency. Additional critical requirements include chemical and thermal stability, the elimination of hazardous heavy metals and cost-effective production methods. Other requirement is chemical stability, thermal stability and heavy metal free as well as cheap in the production. In the case of electrochemical devices such as batteries, fuel cell other parameters are also important: match of thermal expansion coefficient between components, protection before long time oxidation and cyclic change of temperature. These include matching the thermal expansion coefficients of various components to prevent structural degradation, ensuring long-term resistance to oxidation and maintaining stability under cyclic temperature variations.

#### 4.1.2. Anodes

Lithium-ion batteries (LIBs) have solidified their position as the leading energy storage technology, offering unmatched efficiency and versatility for both portable electronics and stationary power systems.

The anodisation of titanium presents a promising technique for fabricating titania films, offering significant potential as anode materials in lithium-ion batteries. By leveraging anodised titania, researchers can develop high-efficiency, scalable battery solutions, paving the way for advancements in compact and high-performance energy storage technologies. Alloying materials such as Si, germanium Ge, Sn and Sb have emerged as high-performance anode candidates for next-generation LIBs and sodium-ion batteries (SIBs). Their exceptional capacity, optimised working voltage and natural abundance make them highly attractive for advancing energy storage technologies. Emerging anode materials for LIBs and SIBs can be classified into three categories based on their reaction mechanisms with Li and Na ions: conversion, intercalation and alloying. Among these, intercalating graphite remains the most widely utilised anode material in LIBs due to its stable structure, excellent cycle life and high conductivity. Porous alloys exhibit exceptional potential for Li-ion and Na-ion storage, leveraging the structural benefits of both nano- and micromaterials. Recent advancements focus on composition refinement, optimised structural design and targeted modifications to further improve electrochemical performance by anodisation.

The anodisation of magnesium at potentials ranging from 3.0 to 6.0 volts reveals a pronounced oxidation process on the surface, resulting in a less favourable dynamic potential pattern. This suggests that higher anodising potentials may negatively impact the structural and electrochemical characteristics of the material. In contrast, anodised magnesium at 1.5 volts demonstrates satisfactory discharge performance, attributed to its optimised microstructure and well-balanced dynamic potential characteristics. This highlights the importance of precise anodising conditions in tailoring the electrochemical behaviour of magnesium-based electrodes for improved energy storage and efficiency [289].

The rapid advancement of flexible electronic devices has driven the development of innovative flexible materials, particularly for next-generation flexible battery technologies. Among these materials, stainless mesh stands out due to its adaptability and suitability for electrode fabrication. Composed of approximately 70% iron, stainless mesh can undergo oxidation to form Fe_2_O_3_, a widely utilised component in battery electrodes.

#### 4.1.3. Cathodes

Ongoing research focuses on improving cathode material stability, optimizing electrolyte formulations and incorporating protective coatings to minimise ion dissolution and extend battery cycle life. The degradation of cathode materials in lithium-ion batteries leads to the release of transition metal ions into the electrolyte, significantly impacting battery performance. These ions contribute to capacity fade and eventual cell failure by interfering with electrochemical reactions and destabilizing the electrode structure.

In lithium-ion batteries, the anode is typically composed of a graphite-based slurry, which is applied to a copper foil current collector to facilitate efficient electron flow. Meanwhile, the cathode consists of transition metal oxides, including LiCoO_2_ (LCO), LiMn_2_O_4_ (LMO), LiNiMnCoO_2_ (NMC), LiNiCoAlO_2_ (NCA) or lithium iron phosphate (LFP) slurry, all of which are coated onto an aluminium foil current collector for enhanced conductivity and stability.

Recent advancements in cathode materials have concentrated on optimizing the electrochemical performance of NMC and NCA compositions. These materials are being refined to improve energy density, cycle stability and thermal safety, making them integral to state-of-the-art battery technologies. Research efforts continue to explore structural modifications and doping strategies aimed at further enhancing the longevity and efficiency of these cathode materials.

For efficient oxygen reduction in aluminium-air batteries, the cathode material must exhibit a porous structure, enabling rapid electron absorption. The two-step anodising process plays a crucial role in forming the aluminium pores necessary for optimised electrode performance. The first anodising step, conducted over six hours, was followed by an etching process lasting 50 min, ensuring controlled pore formation. Subsequently, the second anodising step was carried out for three hours, refining the nanoporous structure and enhancing the cathode’s electrochemical characteristics. These meticulously designed anodising conditions aim to maximise electron transport, improve oxygen accessibility and ultimately enhance the battery’s overall energy conversion efficiency [290].

#### 4.1.4. Interconnectors

The anodisation of aluminium is a well-established technique for producing nanoporous insulating films, enabling a wide range of applications, particularly as interconnections for integrated circuits. Interconnectors play a vital role in fuel cell stacks by electrically linking the anode of one cell to the cathode of the neighbouring cell. In addition to establishing electrical continuity, they act as physical barriers that prevent mixing of the fuel and oxidant gases, thereby maintaining efficient and safe operation [291,292]. These components are engineered from materials that offer high electronic conductivity, resistance to oxidation, gas impermeability and chemical compatibility with other elements of the fuel cell system [293,294]. These films are typically fabricated through galvanostatic anodising in aqueous acidic solutions, using current densities between 6–10 mA/cm^2^ at anodic voltages ranging from 40–200 V to achieve a controlled porous structure [295]. A major challenge in fuel cell technology lies in achieving reliable sealing between the interconnect plates and adjacent flat components. This insufficient sealing compromises device integrity and significantly reduces operational lifespan. As a result, recent research has increasingly concentrated on engineering planar-type SOFC systems designed to function efficiently at lower operating temperatures ranging from 873 to 1073 K, aiming to enhance both performance and long-term durability through improved manufacturing techniques and materials innovation [296].

Recent advancements have demonstrated the viability of anodising in the formation of self-planarised aluminium interconnections for CMOS ultra-large-scale integration, achieving a minimal resolution of 1.2 μm. This progress highlights anodised aluminium’s role in enhancing circuit efficiency, precision and material stability, making it an indispensable component in modern semiconductor technologies.

### 4.2. Surface Engineering for Mechanical and Optical Properties

Structural colour arises from fundamental optical phenomena, including thin-film interference, light scattering and diffraction, rather than pigmentation. Thin-film interference occurs when light waves reflect off multiple layers of a thin material, creating iridescent colours. Light scattering, influenced by particle size and arrangement, generates vivid effects like those observed in the sky’s blue hue or opal gemstones. Diffraction, the bending and spreading of light waves around structures, produces spectral patterns, similar phenomena are observed in anodised materials [297].

Pure Nb_2_O_5_ exhibits a high refractive index of approximately 2.34. When the surface is modified with Nb_2_O_5_ using the anodisation method, the material demonstrates an increased refractive index of 2.46 ± 1%, indicating a notable enhancement in optical properties [298]. Nb_2_O_5_ is considered a transparent oxide semiconductor, demonstrating remarkable optical clarity in the spectrum due to its wide band gap. Since its initial study in 1980, Nb_2_O_5_ electrochromic properties have gained significant attention, owing to its robust chemical stability and resistance to acid and alkali degradation. Advancements in fabrication techniques, such as the two-step solution method, have enabled the production of single-crystal porous Nb_2_O_5_ nanotubes and nanorods, with band gap values of 3.97 eV and 3.72 eV, respectively. The observed blue shift in porous nanotubes compared to solid nanorods is attributed to quantum confinement effects, highlighting the profound influence of material morphology on electronic behaviour. Additionally, grain size has been identified as a crucial factor affecting local coordination and absorption edge energy. That means a tailorable electronic structure based on compositional modifications.

Extensive research has been conducted on the band gap properties of crystalline Nb_2_O_5_ nanofibres; particularly, H-Nb_2_O_5_, O-Nb_2_O_5_ and M-Nb_2_O_5_. H-Nb_2_O_5_ possess a highly ordered structure, characterised by a distinct division into structural blocks [299]. M-Nb_2_O_5_ is classified within the tetragonal crystal system, specifically characterised by the space group I4/mmm and O-Nb_2_O_5_ posses orthorhombic symmetry. Measurements indicate band gaps of 3.85, 3.77 and 3.79 eV, respectively [300]. Complementary studies have reported band gap values of 3.4 eV for orthorhombic Nb_2_O_5_ and 3.1 eV for its monoclinic phase, further illustrating the impact of crystallinity and doping variations on optical absorption [301]. In the same paper, the authors show that the introduction of germanium into monoclinic Nb_2_O_5_ changed the absorption edge from 3.1 to 3.35 eV.

Furthermore, its electrochromic nature enables dynamic optical modulation, transitioning from high transparency (≈85%) to significantly reduced transmittance (<10%) from UV to near-infrared spectra. Depending on its structural characteristics, Nb_2_O_5_ can undergo colour shifts from blue to brown, further enhancing its utility in electrochromic applications. This collective research underscores the versatile optoelectronic capabilities of Nb_2_O_5_, positioning it as a material of high interest for next-generation energy storage, sensing and display technologies.

Zinc oxide has garnered significant attention as a highly promising material for electronic applications. This interest stems from its wide direct bandgap of 3.37 eV and substantial binding energy of 60 meV. N-type ZnO thin films exhibit exceptional visible light transmission, outstanding stability and a remarkable combination of optical, electrical, piezoelectric and thermal characteristics. A key property of ZnO is its ability to generate photoexcited electron-hole pairs upon exposure to light, leading to visible luminescence even under different UV excitation wavelengths. This phenomenon occurs due to the formation of various types of defects. Moreover, native point defects in doped and undoped ZnO thin films play a crucial role in shaping their optical and electrical properties. These features have already enabled their use in devices such as gas sensors, ultrasonic oscillators and transparent electrodes for solar cells. The advantageous electrical and optical properties of ZnO thin layers position them as an ideal choice for a wide range of optoelectronic applications [302,303,304].

Metallic Cu possesses remarkable optical characteristics that make it highly valuable across various applications. In its pure form, copper exhibits a distinct reddish-orange hue, though its appearance can shift to brown or gray depending on surface treatment and the presence of other elements or compounds. Its bright metallic cluster enhances its ability to reflect light efficiently, giving it a shiny, polished finish. Unlike transparent materials, copper completely blocks visible light, preventing light from passing through. However, it is exceptionally reflective, effectively bouncing light off its surface, making it an ideal choice for applications requiring high reflectivity, such as mirrors and optical coatings. Oxide thin films, nanostructures and core–shell architectures have garnered significant interest as optical limiters, primarily due to their ability to integrate multiple optical limiting mechanisms

Copper oxide exists primarily in two stable forms: CuO and Cu_2_O, both of which exhibit remarkable electrical and optical properties. CuO appears dark brown or black, while Cu_2_O has a distinct yellow-to-red colouration. CuO possesses a smaller bandgap than Cu_2_O, enhancing its effectiveness in photon detection and optical switching applications, particularly within the visible and near-infrared spectra. Structurally, Cu_2_O adopts a cubic form with a bandgap ranging from 2.0 to 2.6 eV, while CuO features a monoclinic configuration—with three axes of unequal length, two of which are perpendicular—boasting a bandgap between 1.3 and 2.2 eV. Due to its high electron mobility and strong optical absorption in the visible range, CuO holds considerable potential for solar cell applications. Additionally, it offers high electrical conductivity. Copper oxides function as p-type semiconductors and are frequently paired with n-type counterparts like zinc oxide to optimise performance.

Magnesium oxide has gained considerable attention due to its exceptional properties, including a high dielectric constant (ca. 9.8), a wide bandgap of 7.3–7.8 eV and an impressive breakdown field of 12 MV/cm. These characteristics make it a highly attractive material for advanced electronic and optical applications.

The thick films obtained at substrate temperatures higher than 663 K have quite a good transmittance (80–90%) in a wide wavelength range. The optical band gap values were found to be E_g_ = 3.64–3.70 eV, which is much lower than the band gap of bulk material. The observed optical band gap of the films increased with the substrate temperature. The PL spectra show emission peaks at ≈412 nm (3.00 eV) and at ≈524 nm (2.38 eV), which were attributed to positively and negatively charged F^−^ centres [305,306,307].

Magnesium oxide nanostructures have been effectively utilised as ultra-thin shells on the surfaces of metal oxides such as SiO_2_, ZnO and TiO_2_, significantly enhancing the efficiency of dye-sensitised solar cells. These coatings improve electron transport, stability and light absorption, making them valuable in photovoltaic applications. Additionally, magnesium hydroxide (Mg(OH)_2_) is widely recognised for its non-toxicity, exceptional long-term stability and strong corrosion resistance, making it an optimal material for various industrial and environmental applications, including flame retardants, wastewater treatment and pharmaceutical formulations. Other investigations show that the optical band gap of Mg(OH)_2_ nanostructures, MgO nanoflakes and MgO/Mg(OH)_2_ nanocomposite have values of 3.5, 3.46 and 4.5 eV, respectively [308].

Anodically grown Ta_2_O_5_ films, derived from e-beam deposited tantalum layers on BK7 glass substrates, exhibit exceptional optical transparency across a vast wavelength spectrum, including the ultraviolet range.

The films exhibit high transparency, maintaining an average transmittance exceeding 80% across the wavelength range of 300 to 800 nm. This indicates their suitability for optical applications requiring minimal light absorption. However, below 300 nm, the transmittance declines rapidly as the wavelength decreases, suggesting significant optical absorption in the ultraviolet region. The optical transmittance of the thin films ranges from approximately 75% to 95%, influenced by thin film interference effects. This phenomenon arises from the superposition of light waves reflected from both surfaces of the film, leading to noticeable peaks and valleys in the spectra. These variations are attributed to constructive interference, which enhances transmission at certain wavelengths and destructive interference, which reduces it. Such interference plays a crucial role in optimizing optical coatings, anti-reflective layers and wavelength-selective filters, making it a key factor in advanced photonic applications.

The influence of anodising bath composition and pH on the photoelectrochemical behaviour of tantalum-based anodic films was systematically examined across varying formation voltages, directly affecting film thickness. An estimated band gap of ≈4.1 eV was observed for films anodised at 5 and 50 V, utilizing NaOH and (NH_4_)_2_SO_4_/H_2_SO_4_ solutions. Direct bandgap and indirect bandgap of the tantalum oxide films were measured as 4.05–4.18 eV and 4.53–4.66 eV, respectively [309].

This finding highlights the impact of electrolyte conditions on the optical and electronic properties of tantalum oxide films, offering insights into their potential applications in photoelectrochemical systems and energy conversion technologies [310].

The Ta_2_O_5_ inverse opal thin films and powders exhibit structural colouration across both ultraviolet and visible wavelengths, demonstrating well-defined photonic band gap properties. The photonic band gap position along the [111] crystallographic direction increases proportionally with macropore diameter and the refractive index of the medium filling these pores, aligning precisely with a modified Bragg’s law expression [311].

This low absorbent nature makes them highly suitable for optical coatings, photonics applications and advanced laser systems, where minimal light absorption is crucial for enhanced efficiency and performance.

Porous structure of the anodised Fe_2_O_3_ membranes and layers can be adjusted by varying anodisation parameters like current waveform, time and temperature, leading to different structural colours visible in the film. Typical hematite (*α*-Fe_2_O_3_) has a band gap in the range of 1.9 to 2.3 eV, which is suitable for absorbing a portion of the solar spectrum and has potential in photoelectrochemical applications. The optical properties of periodic Fe_2_O_3_ films can be effectively tailored by modifying anodisation parameters, allowing precise control over their light absorption, reflection and transmission characteristics [312].

An increase in grain size during anodisation is anticipated to enhance the conductivity of *α*-Fe_2_O_3_ by minimizing the recombination rate of holes and electrons. This improvement stems from the refined electrode surface achieved through anodisation, which facilitates more efficient charge transport. By optimizing grain structure, the material exhibits better electronic pathways, leading to increased performance in photoelectrochemical and energy storage applications [313].

The effective refractive index of the material exhibits a gradual increase during the anodisation process, ranging from 1.9 to 2.13 as anodising time progresses. This trend suggests a modification in the material’s optical density, likely influenced by structural changes such as pore formation, oxide layer thickening or compositional adjustments over time [297].

### 4.3. Biomedical Applications

Although it is generally considered that aluminium oxide is biologically inert, it is hazardous to some species. Doskocz and colleagues [314] observed the inhibition growth of *Pseudomonas putida* bacteria when in contact with nanoparticles of Al_2_O_3_. Moreover, the toxicity was evaluated to be very and slightly toxic (according to European standards [315]) for <50 nm nanoparticles and bulk, respectively.

Toxicity of anodised Al_2_O_3_ against *Escherichia coli* was observed by Schabikowski et al. [316]. Since anodisation of aluminium is typically made with the use of toxic precursors, the group analysed matrices without the use of chromium (used in that case for the removal of the first-step AAO) and chloride compounds as well with a similar effect. This work was extended by adding copper ions in different concentrations via chemical functionalisation [317]. The concentration changes were realised with the use of “spacers” which are functional groups unable to attach copper ions and block access to the surface. Copper was anchored on the surface of AAO via propyl copper phosphonate active units. Functional materials with controllable concentrations are mostly based on the use of an inert matrix and appropriately distributed anchoring groups that are able to capture the relevant molecules and control their position [318,319]. This approach makes it possible to obtain materials with unprecedented magnetic, biological or electronic properties [320,321,322]. The presence of surface hydroxyl groups is also important, as this allows for easy grafting with dedicated anchoring groups and control of their distribution [323].

Schabikowski et al. [317] tested both pristine and functionalised matrices against K12, R2, R3 and R4 strains of *E. coli* and the results show that the antibacterial performance approaches those of some antibiotics (i.e., ciprofloxacin, bleomycin and cloxacillin). However, the functionalisation did not improve the toxicity in this particular case dramatically and it seems the main contributor is the AAO matrix itself.

Such modern biocidal materials that can help overcome the problem of antibiotic resistance are experiencing a renaissance [324].

Nanotubes, thanks to their open and ordered structure, allow us to introduce appropriate substances along with the implant, making it possible to improve properties related to bioactivity or biocompatibility. Nanotubes can be filled with various particles. There are many ways of modification, among which the most common are dip coating and spin coating. These provide a uniform layer to be achieved. Immersion coating involves introducing the substrate into the solution, then gravity drainage is performed and, finally, the solvent is evaporated. In spin coating, the substrate is made to rotate and then coated with the solution. In the final stage of the process, the solvent is evaporated. The process of functionalisation of nanotubes with biomolecules makes it possible to increase compatibility with tissues or give the material additional properties. The biomolecules shown in Table 1, such as proteins, peptides or polysaccharides, affect bone regeneration processes. Chitosan, thanks to its bioadhesive properties, significantly improves the deposition of bone cells on the implant surface. For functionalisation, in addition to biomolecules, nanotubes can also be filled with nanoparticles of metal. Gold nanoparticles can form a hydrophobic coating [325]. Zinc is a very important element found in the human body. It can promote DNA synthesis and improve the function of osteoblasts [325]. Copper has a strong effect on the healing of burns and can also prevent infections. According to studies, it has 100% effectiveness against *Escherichia coli* bacteria [325]. The geometry of the nanotube layers creates the possibility of storing drugs in them (as capsules) and then releasing them in a controlled manner in the body or onto biomedical implants. Shrestha et al. [326] demonstrated that TiO_2_ nanotubes can be filled with magnetic Fe_3_O_4_ particles and then magnetically directed to desired locations. Such TiO_2_ nanotubes can be easily coated with drugs using appropriate linkers [327].

### 4.4. Other Applications

Anodisation remains a key technique for fabricating nanostructured ZnO materials with tailored properties for diverse applications. The structural diversity and inherently high surface-to-volume ratios of these nanomaterials have sparked significant interest in their application across multiple fields. Search work focuses on the application as a semiconductor, corrosion protection, adhesion promoter, abrasion protection or antibacterial surfaces. These include high-surface-area electrodes for batteries, photocatalysis for water treatment, carbon dioxide reduction, water splitting, gas sensing, infrared detection and the development of functional surfaces with enhanced wettability properties. AZO layers play a crucial role as protective coatings, mainly for steel components commonly applied to fencing structures, car exteriors and fastening elements such as nails, screws and nuts [128,129]. Additionally, zinc oxide demonstrates remarkable electronic properties making it a viable component of: light-discharging diodes [328], solar cells [329], gas detector [330], water purification [133,139] and photo-catalysts [331]. These properties make ZnO/AAO a suitable electronic material in various fields as bio-molecule sensor, ultraviolet detector, light-emitting diode, chemical and gas sensor, solar cell, microelectronics and optoelectronic [128,133,332,333,334,335,336,337,338,339,340,341,342]. ZnO surfaces exhibit antibacterial characteristics, making them valuable for use in biotechnological and biomedical fields [343].

Copper ions, continuously released from the surface, interact with viruses, bacterial cells and other microorganisms, effectively neutralising them on treated surfaces [344]. When applied as coatings on aluminium substrates, copper not only adds decorative appeal but, more importantly, also delivers a cost-effective and efficient broad-spectrum antibacterial and antiviral functionality—making it particularly suitable for aluminium alloys in public-facing applications. Copper-filled anodised aluminium oxide (Cu-filled AAO) is proposed as a promising base material for interconnection between different layers to be used in energy storage and lab-on the chip experiments [205]. A complementary experiment highlights its potential as a low-temperature bonding material suitable for three-dimensional packaging. This material is fabricated by introducing copper into the isolated nanopores of an AAO matrix, which is produced through anodic oxidation of aluminium. The resulting structure features copper nanofilaments protruding slightly from the AAO surface—referred to as “Cu nano-nails”—enabling effective bonding to copper pads.

Mg alloys has been studied as materials for implants in medical applications since the XIX century. The low corrosion resistance was considered an advantage to a certain extent since magnesium is required in the human body and its toxic dose is currently unknown. Thus, an Mg alloy implant coated with a coating improving biocompatibility and corrosion resistance is considered a good candidate for degradable material compared to polymers as overviewed by Hornberger et al. [345].

In practical terms, anodised tantalum oxide (ATO) is frequently employed in capacitors and as a protective coating for chemical and optical equipment [45]. Its enhanced bioactivity has additionally promoted its adoption in various medical devices [219,266]. Nanostructured ATO materials are gaining interest for use as catalysts, as supports for fuel catalysts, as waveguides, in biomedicine for photoelectrochemical applications.

Its dielectric constant is near 25 which renders it especially attractive for electronic and sensor applications [219,222,346,347,348,349]. Moreover, anodising tantalum produces a dense Ta_2_O_5_ layer known for its high corrosion resistance and inert behaviour which safeguard the material against chemical attack [350,351].

The resulting ATO membranes show promise in numerous applications—including use in electrolytic capacitors, photocatalysis, molecular separation, Bragg-type sensors, dielectric spacers, sensing platforms and nanolithography templates—and they can be transferred onto substrates such as silicon wafers or glass slides to extend their deployment in optical, sensing and catalytic devices.

## 5. Limitations and Challenges of Anodisation

The anodisation process obviously has its limitations. Being an electrochemical process, it suffers from the typical issues those processes have: it is sensitive to, among others, the changes of the electrolyte conditions (temperature, pH or concentration fluctuations), voltage and substrate temperature. Maintaining constant homogeneous conditions is especially important in the case of the fabrication of highly ordered pore arrangement. Good arrangement comes with regularly shaped pores. Fluctuations in electrolyte parameters often lead to irregular pore morphology or even their merging, which results in the deformation of the global mesh of pores. The temperature of the electrolyte is especially endangered because the process is highly exothermic. Elevated temperatures may compromise the integrity of the oxide layer by accelerating chemical dissolution, causing pore deformation and increasing the risk of dielectric breakdown. Non-homogeneous temperature distribution may cause internal stress and thermal expansion mismatch, resulting in cracking or delamination of the oxide layer. A rough surface of a substrate introduces other deviations in morphological conditions. Uneven current distribution in the substrate may cause non-uniform oxide thickness. Trace elements present in the substrate and an electrolyte may cause non-homogeneous dissolution, which disturbs the process, and even chemically contaminate the oxide layer. The contamination may then influence chemical functionalisation, which often requires specific chemical conditions present on the surface. Finally, reproducing regular nanopore structures on a larger area becomes more difficult with the increasing size of the substrate, causing reproducibility and scalability problems on an industrial scale.

Arguably, some applications, such as corrosion resistance, hardness or biocompatibility improvements, do not require highly ordered anodised matrices. However, for advanced magnetic, electronic or optical purposes, the close-to-ideal arrangement is preferred.

To combat the difficulties of reproducible fabrication of highly ordered matrices, several steps are typically applied. Using chemicals and substrates of high purity helps with obtaining homogeneous and less-chemically contaminated products. Substrate pre-treatment by mechanical or electrochemical surface polishing provides more uniform starting morphological conditions. A common strategy is to calcinate the metal before anodisation to release any stress that might have been caused my machining or other factory processes. A breakthrough was made in this matter by Masuda et al. [54]. The two-step anodisation Masuda developed provides a step further from polishing, namely introducing pre-arranged placement for the pores to grow in a more orderly manner. If needed, the intermediate anodisation steps could be extended to three or more for better starting morphology. Another approach is pre-patterning a substrate by means of lithography. Here, we can distinguish a few methods. A nanoimprint lithography method utilises a pre-patterned stamp which indents the pattern directly onto the surface of the metal or indirectly through means of soft resins. Focused-ion beam lithography provides means of pre-patterning even smaller shapes on the surface. Interference and electron beam lithographies imprint patterns into a resist which is then then developed and used for etching the substrate. All these methods, while time-consuming and difficult to apply on larger areas, provide additional benefit: arrangements other than the hexagonal ordering. Finally, colloidal lithography utilises self-assembling monolayers of polystyrene or similar nanospheres as masks.

Careful control of anodisation parameters prevents unwanted fluctuations. This is especially important for thermal control of the reactor. The control of the temperature is often maintained by external cooling system, such as cooling baths, coolant circulation or Peltier cooling plates near the substrate. Mechanical or magnetic stirring may also be implemented to maintain more uniform temperature of an electrolyte. An electrolyte can be cooled prior to the process to start the process as far from the critical temperature as possible. If those precautions are not sufficient, a pulsed anodisation can be applied to provide more time for heat dissipation in between voltage applications.

Anodised membranes are fragile. A free-standing membrane, separated from the metallic substrate, may easily crack under stress. Its strength increases with the thickness of a membrane, thus thin free-standing membranes may not be suitable for applications where a force is applied. Opening pores on the barrier layer by mechanical polishing or chemical etching may also introduce stress and defects further decreasing mechanical durability.

To alleviate the problem, anodised membranes are used with the metallic substrate as a support when possible. In other cases, healing agents can be incorporated to repair mechanical damage and or or hybrid coatings with the addition of polymeric protection layer [352]. Annealing a membrane after the process is also utilised to improve the crystallinity and mechanical stability of oxide films. Finally, fabricating a thicker membrane may improve the durability.

Despite its advantages, chemical functionalisation of anodised membranes introduces potential complications. Achieving uniform functional group distribution over the membrane may be challenging. Membranes with extremely small pores and those which functional precursor does not wet well are especially prone to variations in concentrations. Chemical functionalisation generally offers good resistance of functional groups to mechanical stress, especially when compared to non-chemical (physical) coatings. Nonetheless, extreme operating conditions can still lead to the degradation of chemical groups.

## 6. Conclusions

Anodisation evolved tremendously from its humble beginnings of corrosion protection technique for metals. This review has demonstrated how the process enables fabrication of tailored coatings of wide range of metals with tailored morphologies, porosities and compositions–structures that can be finely controlled by precise tuning of electrochemical parameters and electrolyte composition.

Targeted chemical and molecular functionalisation expands the functional scope of these matrices even further. Whether through doping, surface grafting, layer-by-layer assembly or nanoparticle incorporation, these post-anodisation modifications open new directions for tailoring surface properties such as wettability, biocompatibility, catalytic activity, antibacterial performance and responsiveness to external stimuli. By combining the structural precision of anodisation with the chemical selectivity of functionalisation, researchers are now developing materials with entirely new functionalities that reach far beyond corrosion protection or simple structural coatings.

Future research should focus on refining anodising protocols to achieve greater structural uniformity and enhance the functional properties of ZnO for optoelectronic, sensing and biomedical applications.

A critical challenge in the anodisation of aluminium alloys lies in the presence of alloying elements such as silicon, iron and copper. These elements can precipitate as intermetallic compounds or secondary phase particles, which disrupt the uniformity of the oxide layer formation and adversely impact the overall anodisation behaviour.

To counteract these effects, the anodisation parameters—including electrolyte composition, operating temperature and applied current density—must be meticulously optimised. These adjustments can partially mitigate the negative influence exerted by structural heterogeneities and enhance oxide layer performance.

The anodisation process is deeply intertwined with the alloy’s microstructural characteristics. A comprehensive understanding of this relationship is essential to achieve controlled oxide growth and consistent outcomes. Notably, electrochemical inhomogeneities within the substrate can lead to directional progression of the oxide layer. This phenomenon tends to follow pathways of minimal electrical resistance, resulting in non-uniform coating thickness and morphology.

It is essential to develop manufacturing techniques that are environmentally sustainable, time-efficient, energy-conserving and capable of delivering reproducible results while complying with established industry standards.

Moreover, achieving precise colour uniformity in anodised coatings presents another significant limitation. Variations in microstructure and localised oxide layer properties contribute to colour mismatch and colour fading is frequently observed, particularly under prolonged exposure to environmental stressors.

Recent advances in additive manufacturing have introduced novel capabilities for integrating complex polymer–metal interfaces. Through 3D printing techniques, it is possible to fabricate intricate polymer joints directly onto anodised aluminium substrates—eliminating the need for traditional mold-based manufacturing and enabling efficient, design-flexible production of hybrid components.

This review highlights how the synergy between nanostructured anodic architectures and chemical modification can unlock applications in biomedicine, energy storage, sensing, photocatalysis and environmental technologies. As research advances, further optimisation of functionalisation techniques and the exploration of novel anodisable substrates will continue to broaden the technological potential of anodised materials. The field is well-positioned to contribute to next-generation functional platforms across a wide set of disciplines.

## Figures and Tables

**Table 1:** The functions of various biomolecules used for functionalisation.

Biomolecules	Functions
Bone Morphogenetic Protein-2 (BMP-2)	Promotes the ability of bone marrow-derived mesenchymal stem cells to differentiate into osteoblasts in vitro
Chitosan	Enhances drug elution, osteoblast adhesion and strengthens bone integration
Polydopamine	Prolongs drug release duration and maintains constant release kinetics
Quercetin	Loads into TNTs and releases in the environment as an alternative for treating postoperative infections, inflammation and promoting faster healing with better integration
Trehalose	Has osteogenic potential and anti-inflammatory properties
Gelatin	Improves osteoblast adhesion and propagation and serves as a drug release-controlling coating
Hemoglobin	Detects hydrogen peroxide
Uricase (Urate Oxidase)	Detects uric acid
Glucose Oxidase	Detects glucose
Polycaprolactone	Improves the solubility and flexibility of nanotubes, enhancing their biocompatibility
Antimicrobial Peptides	Exhibit antimicrobial activity
Osteogenic Growth Peptide	Enhances osteoblast differentiation
Gly-Arg-Gly-Asp-Ser Peptide	Enhances cell adhesion and increases cell spreading and proliferation
Arg-Gly-Asp Peptide	Enhances adhesion of bone marrow stem cells (BMSCs) and significantly improves the expression of osteogenic genes in BMSCs
Lys-Arg-Ser-Arg Peptide	Enhances preosteoblast adhesion and osteogenic gene expression on TNTs
Palmitoyl-oleoyl-phosphatidylcholine	Used as a barrier for controlled and sustained drug release
Epidermal Growth Factor	Promotes MSC proliferation and prevents cell apoptosis
Small Interfering RNA	Targets tumor necrosis factor-alpha (TNF-*α*)

## Data Availability

No new data were created or analysed in this study.

## Abbreviations

The following abbreviations are used in this manuscript:

AAO	anodic aluminium oxide
ATO	anodic tantalum oxide
AZIB	aqueous rechargeable zinc-ion battery
CuNPA	copper nanopillar array
DMSO	dimethyl sulphoxide
DSSCs	dye-sensitised solar cells
EG	ethylene glycol
LIB	lithium-ion batteries
NRs	nanorods
PAA	polyacrylic acid
PECD	pulse electrochemical deposition
PEO	plasma electrolytic oxidation
SIB	sodium-ion batteries
TNA	TiO_2_ nanotube array
